# Supplementary material for: Recovery of europium from E-waste using redox active tetrathiotungstate ligands
Source: Nat Commun. 2024 Jun 3;15:4577. doi: 10.1038/s41467-024-48733-z (PMC11148158; doi:10.1038/s41467-024-48733-z)
Supplement: Supplementary file 1 — Supplementary Information [file 41467_2024_48733_MOESM1_ESM.pdf]

Recovery of Europium from E-Waste Using Redox Active Tetrathiotungstate Ligands

Marie A. Perrin[1], Paul Dutheil[1,2,3], Michael Wörle[1] and Victor Mougel[1*]

[1]Department of Chemistry and Applied Biosciences, ETH Zürich, Vladimir-Prelog-Weg 1-5, 8093 Zürich, Switzerland

[2]Laboratory of Radiochemistry, Nuclear Energy and Safety Division, Paul Scherrer Institute, Forschungsstrasse 111, Villigen PSI, CH-5232, Switzerland

[3]Department of Radiation Safety and Security, Paul Scherrer Institute, Forschungsstrasse 111, Villigen PSI, CH-5232, Switzerland

*Corresponding author. E-mail: mougelv@ethz.ch

Supplementary Information

Table of Contents:

A. Supplementary Methods

1. GENERAL CONSIDERATIONS 5
2. SYNTHESIS 8
   - 2.1 SYNTHESIS OF $(NH_4)_2WS_4$ 8
   - 2.2 SYNTHESIS OF $(NEt_4)_2WS_4$ 8
   - 2.3 SYNTHESIS OF $Eu(OTf)_3$ 8
   - 2.4 SYNTHESIS OF $Y(OTf)_3$ 8
   - 2.5 SYNTHESIS OF $[NEt_4]_2[Eu^{II}(WS_4)_2]$ (1) 9
   - 2.6 SYNTHESIS OF $[NEt_4]_3[Eu^{III}(MeCN)_2(WS_4)_3]\cdot MeCN$ (2) 9
   - 2.7 SYNTHESIS OF $[NEt_4]_3[Y^{III}(MeCN)(WS_4)_3]$ (3) 10
   - 2.8 SEPARATION OF EU FROM 1:1 EU/Y MODEL MIXTURE 10
   - 2.9 SEPARATION OF EU FROM 1:14 (WT%) EU/Y MODEL MIXTURE 10
   - 2.10 EXTRACTION OF LAMP PHOSPHOR 10
   - 2.11 SEPARATION OF EU FROM LAMP PHOSPHOR 11
   - 2.12 RECOVERY OF EUROPIUM OXIDE FROM COMPLEX 1 11
   - 2.13 RECOVERY OF EUROPIUM OXIDE FROM COMPLEX 1:1 (WT%) EU:Y MODEL MIXTURE 11
3. CYCLIC VOLTAMMETRY 12
4. UV-VIS SPECTROSCOPY 13
5. RAMAN SPECTROSCOPY 16
6. INFRARED SPECTROSCOPY 17
7. X-RAY PHOTOELECTRON SPECTROSCOPY 18
8. X-RAY ABSORPTION SPECTROSCOPY 24
9. SINGLE CRYSTAL X-RAY DIFFRACTION 25
10. PAIR DISTRIBUTION FUNCTION ANALYSIS 29
11. BOND VALENCE SUM ANALYSIS 30
12. EU/Y SEPARATION 31
   - 12.1 DETERMINATION OF ENRICHMENT, SEPARATION FACTORS AND EFFICIENCY 31
   - 12.2 SEPARATION OF EU FROM 1:1 EU/Y MODEL MIXTURE 31
   - 12.3 SEPARATION OF EU FROM 1:14 EU/Y MODEL MIXTURE 32
   - 12.4 EXTRACTION OF LAMP PHOSPHOR 32
   - 12.5 SEPARATION OF EU FROM LAMP PHOSPHOR 32

B. Supplementary References

Index Supplementary Figures:

Supplementary Figure 1: CV spectra of complex 2 (dark blue) and 3 (light blue) (1 mM) in 0.1 M TBAPF6 in MeCN 12

Supplementary Figure 2: CV spectra of Eu(III)/Eu(II) reduction wave in complex **2** (1 mM) in 0.1 M $TBAPF_6$ in MeCN at different scan rates (left); Peak current plotted against the square root of the scan rate for scan rate dependance CV measure for complex **2** (right), with cathodic peak current I_C (square) and anodic peak current I_A (triangle). 12

Supplementary Figure 3: UV-Vis electronic absorption spectrum of $(NH_4)_2WS_4$ in H_2O (light orange) and $(NEt_4)_2WS_4$ in MeCN (dark orange). 13

Supplementary Figure 4: UV-Vis electronic absorption spectrum of **1** (red) and **2** (blue) in MeCN. 13
Supplementary Figure 5: UV-Vis electronic absorption spectrum of **3** (green) in MeCN. 14
Supplementary Figure 6: UV-Vis electronic absorption spectrum of the formation of complex **1** (red) from $(NEt_4)_2WS_4$ (black), via the intermediate formation of 2 (blue). 14
Supplementary Figure 7: UV-Vis electronic absorption spectrum of the $(NH_4)_2WS_4$ (prepared according to section 2.1 (dark orange) in H_2O and the aqueous supernatant obtained according to process 2.12 (light orange). Spectrum were normalized from the 216 nm band intensity. 15
Supplementary Figure 8: Low-energy region of resonance Raman spectra recorded on solution samples at room temperature with 532 nm laser excitation. 16
Supplementary Figure 9: FTIR spectra of europium oxalate: as prepared in this work according to section 2.12 (light orange) and from ref[15] (orange). Graph extracted from the Supplementary information section, Figure S59 therein). 17
Supplementary Figure 10: XPS spectra in the Eu 3*d* region for complex **1.** 18
Supplementary Figure 11: XPS spectra in the S 2p region for complex 1. 18
Supplementary Figure 12: XPS spectra in the W 4f region for complex 1. 19
Supplementary Figure 13: XPS spectra in the Eu 3d region for complex 2. 19
Supplementary Figure 14: XPS spectra in the S 2*p* region for complex **2.** 20
Supplementary Figure 15: XPS spectra in the W 4*f* region for complex **2.** 20
Supplementary Figure 16: XPS spectra in the Eu 3*d* region for complexes **1** and **2**, and for the precipitate and filtrate obtained from the 1:1 (wt%) mixture separation. 21
Supplementary Figure 17: XPS spectra in the Y 3*d* region for the filtrate of the synthesis of complex **1,** complex **3** and for the precipitate and filtrate obtained from the 1:1 (wt%) mixture separation. 22
Supplementary Figure 18: XPS survey spectra of complex **1** (red), europium oxalate (orange) and europium oxide (black) prepared according to section 2.12. A strong F 1*s* peak can be observed in the spectra of the europium oxalate precipitate, and can be assigned to co-precipitated $[NEt_4]$OTf by-product. Nevertheless, this signal vanishes after calcination of the oxide, highlighting that this by-product can be quantitatively thermally decomposed. 23
Supplementary Figure 19: Normalized XANES spectra measured in transmission mode at W L_3-edge for $(NEt_4)_2WS_4$ (black), complexes **1** (red) and **2** (blue). Identical whiteline energy can be determined for all three species. 24
Supplementary Figure 20: Mercury diagram of the solid-state molecular structure of **1**. Hydrogen atoms have been omitted for clarity. ORTEP thermal ellipsoids are shown at the 50% probability level. Selected bond lengths and angles are summarized in Supplementary Table 2. 25
Supplementary Figure 21: Mercury diagram of the solid-state molecular structure of **2**. Hydrogen atoms and co-crystallized MeCN molecules have been omitted for clarity. ORTEP thermal ellipsoids are shown at the 50% probability level. Selected bond lengths and angles are summarized in Supplementary Table 2. 26
Supplementary Figure 22: Mercury diagram of the solid-state molecular structure of **3**. Hydrogen atoms and co-crystallized MeCN molecules have been omitted for clarity. ORTEP thermal ellipsoids are shown at the 50% probability level. Selected bond lengths and angles are summarized in Supplementary Table 2. 26
Supplementary Figure 23: The calculated X-ray pair distribution function G(r) of **1** in the distance interval 1.2-6.3 Å based on single crystal XRD data (grey line) and the experimental PDF of the golden precipitate (red dots). The peaks at 2.19 Å, 3.03 Å and 3.79 Å, correspond to the next neighbor W-S, Eu-S and W-Eu distances (2.19 Å, 3.04 Å and 3.78 Å, respectively) found in the PDF of compound **1**. 29

Index Supplementary Tables:

Supplementary Table 1: Selected binding energies (eV) of Eu 3*d* in complexes **1-2** and in europium oxalate and europium oxide as described in synthesis 2.12. 22
Supplementary Table 2: Selected bond distances and angles for complexes **1-3**. 26

Supplementary Table 3: Refinement parameters for complexes **1-3**. 27
Supplementary Table 4. Bond valence sum for Eu in **1**. 29
Supplementary Table 5. Bond valence sum for W in **1**. 29
Supplementary Table 6. Bond valence sum for W in **2**. 29
Supplementary Table 7. Bond valence sum for W in **3**. 29
Supplementary Table 8. ICP-OES quantification of the Eu/Y quantification in the precipitate (Eu phase) and filtrate (Y phase) from the 1:1 wt% $Eu(OTf)_3$ and $Y(OTf)_3$ mixture and duplicates. 30
Supplementary Table 9. Separation factor from the 1:1 (wt%) Eu:Y model mixtures with different extractants. 30
Supplementary Table 10. ICP-OES quantification of the Eu/Y quantification in the precipitate (Eu phase) and filtrate (Y phase) from the 1:14 wt% $Eu(OTf)_3$ and $Y(OTf)_3$ mixture and duplicates. 31
Supplementary Table 11. ICP-OES quantification of the precipitate (Eu phase) over time from the 1:14 wt% $Eu(OTf)_3$ and $Y(OTf)_3$ mixture and duplicates. 31
Supplementary Table 12. ICP-OES quantification of the Eu and Y in the extracted lamp phosphor (PHILIPS Genie compact fluorescent light bulb (14 W energy saver 230-240 V))....31
Supplementary Table 13. ICP-OES quantification of the Eu/Y quantification in the precipitate (Eu phase) and filtrate (Y phase) from lamp extracts obtained according to the process described in section 2.11. 31

A. Supplementary Methods

1. General Considerations

Materials. Unless stated otherwise, syntheses were carried out under strict inert Argon atmosphere using Schlenk techniques or inside Vigor® gloveboxes. Pentane was stirred over concentrated sulfuric acid, rinsed with aqueous bicarbonate solution and deionized water and dried over calcium chloride beads before being used in a solvent purification system. Diethyl ether and acetonitrile were dried using a Vigor® solvent purification system. Diethyl ether was additionally dried over potassium/benzophenone, distilled, degassed by 3 freeze-pump-thaw cycles and stored over 4 Å molecular sieves for at least 3 days prior to use. Likewise, acetonitrile was degassed by 3 freeze-pump-thaw cycles and stored over 3 Å molecular sieves prior to use.

H_2WO_4 was purchased from Fluka. Europium (III) oxide (Eu_2O_3, 99.99% trace metals basis) was purchased from Acros Organics. Yttrium (III) oxide (Y_2O_3, 99.99%) was purchased from Ventron. Trifluoromethanesulphonic acid (99%) was purchased from Apollo Scientific Ltd. Tetraethylammonium hydroxide (25% in water) was purchased from Acros. $(NH_4)_2WS_4$ and $(NEt_4)_2WS_4$ were synthesized according to literature procedure,[1] using either an H_2S gas bottle (99.5%) from Air Liquide or generating H_2S in situ using a Kipps apparatus filled with FeS fused sticks from Merck Millipore and sulfuric acid (H_2SO_4, 95-98%) from Sigma-Aldrich. Ammonium oxalate (98 %) was purchased from Fluorochem.

Elemental analyses were carried out at the Molecular and Biomolecular Analysis Service (MoBiAS) of ETH Zürich on a LECO TruSpec® Micro spectrometer.

NMR data were recorded on a 200 MHz Bruker Avance II spectrometer at room temperature. ^1H spectra are reported in parts per million (ppm) and are calibrated with respect to the corresponding solvent residual peak.

Magnetic susceptibility measurements in the solid state were carried out on a Gouy Balance (Johnson Matthey).

Cyclic voltammograms were recorded under strictly anaerobic conditions in a conventional three electrode single-compartment cell (20 mL) using glassy carbon as working electrode (diameter 3 mm), a platinum wire as counter-electrode and a silver wire dipped in a 0.01 M solution of $AgNO_3$ in a 0.1 M solution of $TBAPF_6$ in MeCN as a reference electrode. The reference electrode was separated from the cell using a guard filled with the same electrolyte as used in the cell, separated by a Vycor® frit. The potential was controlled by a BioLogic SP-300 potentiostat (Bio-Logic Science Instruments SAS). All potentials were referenced to an internal Fc/Fc^+ standard added in the last step of each experiment. The scan rate was 100 $mV.s^{-1}$ unless otherwise specified. The half-have potentials ($E_{1/2}$) were defined as the weighing average of the corresponding cathodic peak potential (E_c) and anodic peak potential (E_a) for reversible processes. For irreversible processes, the potential at which half of the peak current ($I_c/2$) is reached, has been considered as $E_{1/2}$. In all electrochemical studies 1 mM solution of complex in MeCN was used unless stated otherwise.

UV-Vis electronic absorption data in MeCN were collected on an Agilent Cary 60 UV-Vis Spectro- photometer, connected to a sampling probe (*d*=2 mm) located inside a Glovebox via an optical fiber. Measurements were performed on $1 \cdot 10^{-4}$ M acetonitrile solutions, unless stated otherwise.

Raman spectroscopy data were collected using a Thermo Scientific DXR Smart Raman spectrometer and processed with the OMNIC software. Spectra were obtained using a 532 nm excitation laser wavelength, 10 mW output power and a spectral resolution of 1 cm^{-1}. Samples were measured in NMR tubes sealed with a J. Young valve.

Fourier Transform Infrared spectroscopy data were carried out inside a glovebox using a Nicolet iS5 instrument from Thermo Scientific and processed with the OMNIC software. Samples were diluted using a KBr matrix and pressed into pellets.

X-ray photoelectron spectroscopy (XPS) measurements were performed on a Sigma II instrument (Thermo Electron) equipped with an Alpha 110 hemispherical analyzer. The instrument was operated in large area XPS mode using an Al Kα X-ray source at 200 W. All samples were prepared in an Ar-filled glovebox by loading the sample powder into a home-made sample holder that allows for the samples to be transferred into the FEAL chamber under vacuum without being exposed to the ambient atmosphere. The pressure in the XPS analysis chamber was maintained under 5.0 x 10^{-8} mbar during all measurements. Survey scan spectra were collected up to a binding energy of 1100 eV using a pass energy of 50 eV, a step size of 1 eV, and a dwell time of 50 ms. Narrow region scans were collected using a pass energy of 25 eV, a step size of 0.1 eV, and dwell time of 50 ms. All spectra were calibrated to the C 1s peak at 285 eV. XPS data were analyzed with the CasaXPS software.[2]

X-ray absorption Spectroscopy (XAS) measurements were performed at the SuperXAS X10-DA beamline at the Swiss Light Source (SLS) (PSI, Villigen, Switzerland).[3] The beamline itself operates under constant top-up mode at 2.4 GeV and a ring current of 400 mA. The incident beamline was collimated using a Si-coated mirror (2.9 mrad), monochromatized using a liquid nitrogen cooled channel-cut Si(111) monochromator, and then focused by a Rh-coated double focusing mirror to a spot size of 1 × 0.2 mm at the sample position. The beamline energy was calibrated to the W L_3-edge position (10.207 keV) using a Ta reference foil and to the Eu L_3-edge position (6.977 keV) using an Fe foil. All samples were measured simultaneously with the reference foil in transmission mode using a series of ionization chambers filled with N_2 (1 bar). QuickXAS was used for the measurement of all samples, allowing for the rapid collection of 120 spectra over the course of 180 seconds. The spectra were collected over the duration of the XAS measurement then averaged to yield one workable spectrum. XAS spectra were initially processed using ProXAS,[4] which included encoder analysis, calibration, interpolation, and averaging of the spectra. The averaged spectra were then analyzed using the Demeter software suite,[5,6] which included edge energy calibration, background subtraction, and edge step normalization. The absorption edge energy, E_0, was defined as the peak of the first derivative and served as the origin for the spectra once converted to the photoelectron wave vector, k (i.e. k = 0). The resulting $\chi(k)$ functions were k^2-weighted and then Fourier transformed over a range of 2.7 – 11.2 $Å^{-1}$ for all samples. Samples were pressed as 1 mm thick pellets diluted with boron nitride in a Ar-filled glovebox and then sealed under vacuum until just before the XAS measurements were performed.

X-ray Crystallography data were collected on a Rigaku XtaLAB Synergy-S diffractometer equipped with a HyPix-6000HE detector using. CuKα radiation (λ = 1.54184 Å) at 100 K. After data collection, structures were solved by intrinsic phasing (SHELXT) and refined by full-matrix least-squares procedures on F^2 using SHELXL in the *olex2* program suite.[7-10] All non-hydrogen atoms were refined with anisotropic displacement parameters. The hydrogen atoms were placed in positions of optimized geometry.

X-ray Powder diffraction data suitable for Pair Distribution Function (PDF) analysis were collected of a sample sealed in Mark-tube (0.5 mm diameter) using a Stoe STADI P diffractometer (AgKα1 radiation, λ = 0.55941 Å, curved Ge-monochromator) equipped with a Mythen 4K detector. To reduce noise, the diffractogram with 0.015° step width was binned to a step width of 0.06° and the measured background using an empty Mark-tube was subtracted.

The pair distribution function G(r) was obtained using xPDFsuite (Qmin. = 0.308 $Å^{-1}$, Qmax. = 17.084 $Å^{-1}$, R-poly 1.293).[11] For comparison, a pair distribution function was calculated based on the single crystal data of compound **1** using PDFgui v. 1.1a.[12]

Inductively Coupled Plasma Optical Emission spectroscopy (ICP-OES) measurements were carried out with an Agilent 5110 instrument (Agilent Technologies, Inc.) equipped with a double-pass spray chamber and a SeaSpray concentric glass nebulizer. During the measurements, the instrument was operated in its axial mode, with 0.7 $L{\cdot}min^{-1}$ nebulizer flow (Ar), 12 $l{\cdot}min^{-1}$ plasma flow (Ar), 1 $L.min^{-1}$ auxiliary flow (Ar), and 1.2 kW RF power. The instrument was calibrated externally from single-element standards (1000 mg/L, TraceCERT ®, Merck KGaA, Germany) and their dilutions, allowing Eu and Y to be quantified with the 381.967 nm and 360.074 nm spectral lines, respectively. The samples were prepared by dissolution in analytical grade concentrated HNO_3 and a subsequent dilution with milliQ water (18.2 mΩ). In some samples, yellow WO_3 residue had to be filtered off by a 0.45 µm syringe filter.

2. Synthesis

2.1 Synthesis of $(NH_4)_2WS_4$

$(NH_4)_2WS_4$ was synthesized according to the following modified literature procedure.[1]

The synthesis was performed in a well-ventilated fumehood.

H_2WO_4 (10 g, 40 mmol) was dissolved in aq. NH_3 (25%, 60 mL), and the solution was constantly purged with H_2S, bubbling through the solution via a Teflon canula. While maintaining the constant H_2S purge and stirring the solution, color changes from pale white over lime-green to bright green were observed, and after 1 h, the solution was heated gradually to 60 °C over a period of 3 h, cooled down to room temperature, and stirred overnight. After that time the formation of a yellow solid was observed. The solid product was isolated by filtration, washed with ^{i}PrOH (3 x 25 mL) and Et_2O (3 x 25 mL), and dried *in vacuo* to give a yellow powder (10.91 g, 31.3 mmol, 78%). Elemental analysis found (calc.) % for $H_8N_2WS_4$: H, 2.38 (2.32); N, 8.24 (8.05). UV-Vis (H_2O, $0.9 \cdot 10^{-4}$ M, 1 cm path) λ_{max} (nm) (ϵ ($M^{-1} \cdot cm^{-1}$)): 216 (32 740), 278 (28 448), 393 (19 247).

Note: Excess H_2S gas was quenched using gas-wash bottles in series, filled first with sodium hypochlorite followed by 1M KOH.

2.2 Synthesis of $(NEt_4)_2WS_4$

$(NEt_4)_2WS_4$ was synthesized according to the following modified literature procedure.[1]

$(NH_4)_2WS_4$ (4.9 g, 14 mmol) was dissolved in NEt_4OH (25% in H_2O, 16 mL) and degassed H_2O (20 mL). The solution was subjected to pumping for 2 h while stirring, before ^{i}PrOH (150 mL) was added, resulting in the formation of a bright yellow precipitate, which was allowed to settle at 0 °C. The supernatant was removed by cannula filtration and the solids were washed with ^{i}PrOH (2 x 50 mL) and Et_2O (2 x 50 mL) and dried *in vacuo* overnight. The crude product was extracted with MeCN and dried *in vacuo* overnight, affording the title complex as bright yellow crystals (6.06 g, 10.6 mmol, 75%). Elemental analysis found (calc.) % for $C_{16}H_{40}N_2WS_4$: C, 33.60 (33.56); H, 7.03 (7.04); N, 5.10 (4.89). UV-Vis (MeCN, $1.9 \cdot 10^{-4}$ M, 2 mm path) λ_{max} (nm) (ϵ ($M^{-1} \cdot cm^{-1}$)): 223 (28 844); 284 (25 290); 399 (19 534).

2.3 Synthesis of $Eu(OTf)_3$

$Eu(OTf)_3$ was synthesized according to the following modified literature procedure.[13]

The synthesis was performed under aerobic conditions in a well-ventilated fumehood.

In a 100 mL round-bottomed flask cooled in an ice bath, anhydrous trifluoromethanesulfonic acid (2 mL) was added to miliQ water (2 mL) (*Caution! Exothermic reaction*). The reaction was stirred until fuming stopped. Afterwards, Eu_2O_3 (2.016 g, 5.73 mmol) was added portion wise under strong stirring, and the suspension was heated at 110 °C for 2 h. The reaction was allowed to cool down to room temperature and diluted with water (50 mL). The mixture was filtered to remove the unreacted oxide and the solution was further filtered using Acrodisc® Syringe Filters (0.2 μm, 13 mm) resulted in a clear and colorless solution which was dried under vacuum to afford a white powder. The triflate was dried under vacuum (10^{-5} bar) at 200°C for 24 h prior use. Complete dehydration was confirmed by ^{1}H NMR ^{1}H NMR (200 MHz, CD_3CN): δ [ppm] no signal. ^{19}F NMR (282 MHz, CD_3CN): δ [ppm] –81.90 (s).

2.4 Synthesis of $Y(OTf)_3$

$Y(OTf)_3$ was synthesized according to the following modified literature procedure.[13]

The synthesis was performed under aerobic conditions in a well-ventilated fumehood.

In a 100 mL round-bottomed flask cooled in an ice bath, anhydrous trifluoromethanesulfonic acid (2 mL) was added to miliQ water (2 mL) (*Caution! Exothermic reaction*). The reaction was stirred until fuming stopped. Afterwards, Y_2O_3 (1.30 g, 5.73 mmol) was added portion wise under strong stirring, and the suspension was heated at 110 °C for 2 h. The reaction was allowed to cool down to room temperature and diluted with water (50 mL). The mixture was filtered to remove the unreacted oxide and the solution was further filtered using Acrodisc® Syringe Filters (0.2 μm, 13 mm) resulted in a clear and colorless solution which was dried under vacuum to afford a white powder. The triflate was dried under vacuum (10^{-5} bar) at 200°C for 24 h prior use. Complete dehydration was confirmed by ^{1}H NMR. ^{1}H NMR (200 MHz, CD_3CN): δ [ppm] no signal. ^{19}F NMR (282 MHz, CD_3CN): δ [ppm] –79.26 (br).

2.5 Synthesis of $[NEt_4]_2[Eu^{II}(WS_4)_2]$ (1)

In a 25 mL scintillation vial, $(NEt_4)_2WS_4$ (200 mg, 0.35 mmol, 3 equiv.) was solubilized in 5 mL of MeCN, resulting in a bright yellow solution. $Eu(OTf)_3$ (70 mg, 0.117 mmol, 1 equiv.) was then added, resulting in an immediate color change to dark red. After one hour a large amounts of a golden-brown precipitate could already be observed. The reaction was further stirred at room temperature for 24 h, before the solution was centrifuged and washed with MeCN (3 x 2 mL) (118 mg, 0.113 mmol, 96 % yield). Single crystals suitable for XRD analysis were obtained from the slow diffusion of the reactants at room temperature by layering dilute solutions under ambient conditions. Alternatively, single crystals of 1 could also be obtained by slow vapor diffusion of Et_2O onto a solution of the reactants. UV-Vis (MeCN, $2{\cdot}10^{-4}$ M, 2 mm path) λ_{max} (nm) (ϵ ($M^{-1}{\cdot}cm^{-1}$)): 287 (13 559); 398 (17 952); Elemental analysis found (calc.) % for 1 ($C_{16}H_{40}EuN_2S_8W_2$): C, 18.23 (18.54); H, 3.78 (3.89); N, 2.76 (2.70).

A magnetic susceptibility of 7.2 μ_B was determined at room temperature, in good agreement with the total spin of 7/2 (μ = 7.9 μ_B) expected for Eu(II).[14]

Influence of light and heat here on the formation of (1)

To investigate the impact of external stimuli (heat and light) on the formation of (1), the same reaction than described above was carried out in the absence of light at room temperature and at 60°C. For each reaction (no light and ambient temperature, ambient light and temperature, ambient light and 60 °C) $Eu(OTf)_3$ (11 mg, 0.018 mmol, 1 equiv.) and $(NEt_4)_2WS_4$ (29 mg, 0.050 mmol, 2.7 equiv.) were solubilized in 2 mL of MeCN. To better discriminate the influence of these stimuli, the precipitate formed over the reaction was collected, dried and weighted after 1h stirring. The synthesis under ambient light at room temperature yielded 9.46 mg of 1 were collected (51 % yield), a decreased yield was observed when the reaction is conducted in the dark (6.4 mg of 1 collected, 35 % yield), and an increased yield was determined when the reaction is conducted at 60 °C (15.9 mg of 1 collected, 83 % yield).

2.6 Synthesis of $[NEt_4]_3[Eu^{III}(MeCN)_2(WS_4)_3]{\cdot}MeCN$ (2)

The synthesis was conducted with a minimal exposure to ambient light and always handling solutions cooled at −35 °C.

In a 25 mL scintillation vial, $(NEt_4)_2WS_4$ (200 mg, 0.350 mmol, 3 equiv.) was solubilized in 5 mL of MeCN resulting in a bright yellow solution. The latter was treated with $Eu(OTf)_3$ (70 mg, 0.117 mmol, 1 equiv.) resulting in an immediate color change to dark red. The reaction was kept at −35 °C for 24h before it was layered in the dark with pentane (2 mL) and Et_2O (5 mL) and further stored at −35 °C. After a day, dark red crystals were isolated by pipetting out the supernatant. The large red crystals co-crystalize with small amounts of the fine golden-brown precipitate of 1, but this fine powder can be separated from the larger single crystals of 2 by suspending it in Et_2O (3 x 3 mL). Large red crystals of the title compound obtained after that rinsing step were dried under vacuum (122 mg, 0.077 mmol, 66 % yield). UV-Vis ($1.2{\cdot}10^{-4}$ M MeCN, 2 mm path) λ_{max} (nm) (ϵ ($M^{-1}{\cdot}cm^{-1}$)): 285 (42 147); 398 (41 713); 440 (64 232). $E_{1/2}$ = − 0.55 V (*vs.* Fc/Fc^+) in 0.1 M $TBAPF_6$ in MeCN.

Elemental analysis found (calc.) % for 2·0.5 MeCN ($C_{29}H_{67.5}EuN_{5.5}S_{12}W_3$): C, 22.01 (22.02); H, 4.74 (4.30); N, 4.53 (4.87).

Note that when a dilute (1 mM) solution of 2 was left standing 24h in MeCN under ambient conditions, crystals of 1, suitable for single crystal XRD are formed.

2.7 Synthesis of $[NEt_4]_3[Y^{III}(MeCN)(WS_4)_3]$ (3)

In a 25 mL scintillation vial, $(NEt_4)_2WS_4$ (100 mg, 0.174 mmol, 3 equiv.) was solubilized in 5 mL of MeCN, resulting in a bright yellow solution. $Y(OTf)_3$ (31.2 mg, 0.058 mmol, 1 equiv.) was added as a solid to this solution, resulting in an immediate color change to bright orange. The reaction was stirred at room temperature for 24h before it was layered with pentane (2 mL) and Et_2O (5 mL) and crystallization was set at −35 °C. After a day, red crystals were isolated by pipetting out the supernatant, washed with Et_2O (2 x 2 mL) and dried under vacuum (82 mg, 0.055 mmol, 95 % yield). UV-Vis ($1\cdot10^{-4}$ M MeCN) λ_{max} (nm) (ϵ ($M^{-1}\cdot cm^{-1}$)): 220 (39 335); 283 (51 020); 398 (44 689); 441 (6 079). Elemental analysis found (calc.) % for 3·0.5 MeCN ($C_{27}H_{64.5}N_{4.5}S_{12}W_3Y$): C, 22.23 (21.95); H, 4.81 (4.40); N, 3.90 (4.27).

2.8 Separation of Eu from 1:1 Eu/Y model mixture

In a 20 mL scintillation vial, $(NEt_4)_2WS_4$ (190 mg, 0.336 mmol, 7 equiv.) was solubilized in 10 mL of MeCN, resulting in a bright yellow solution. $Eu(OTf)_3$ (30 mg, 0.050 mmol, 1 equiv.) and $Y(OTf)_3$ (30 mg, 0.056 mmol, 1.1 equiv.) were added as solids to this solution, resulting in an immediate color change to dark red. After one hour a golden-brown precipitate was observed. The reaction was stirred at room temperature for 24h, before the solution was centrifuged to separate the red filtrate from the golden-brown precipitate. The latter was washed with MeCN (2 x 4 mL) and then both phases were dried under vacuum yielding a golden solid (46.6 mg) and a red solid (178.9 mg) which were characterized by ICP-OES (Supplementary Table 8). It should be noted that when the red filtrate is stored at −35 °C, single crystals of complex 3 can be isolated. Elemental analysis found for the golden solid (calc.) % for $C_{16}H_{40}EuN_2S_8W_2$ (Europium phase): C, 18.52 (18.54); H, 3.90 (3.89); N, 2.80 (2.70), in agreement with its assignment as complex 1.

2.9 Separation of Eu from 1:14 (wt%) Eu/Y model mixture

In a 25 mL scintillation vial, $(NEt_4)_2WS_4$ (480 mg, 0.840 mmol, 50 equiv.) was solubilized in 20 mL of MeCN resulting in a bright yellow solution. $Eu(OTf)_3$ (10 mg, 0.016 mmol, 1 equiv.) and $Y(OTf)_3$ (140 mg, 0.261 mmol, 15.6 equiv.) were added as solids to this solution, resulting in an immediate color change to dark red. After one hour stirring at room temperature, a golden-brown precipitate had formed. The reaction was stirred at room temperature for 24h, before the solution was centrifuged to separate the red filtrate from the golden-brown precipitate. The latter was washed with 2 x 4 mL of MeCN and then both phases were dried under vacuum yielding a golden solid (18.5 mg) and red solid (599.8 mg) which were characterized by ICP-OES (Supplementary Table 10). The composition of the golden precipitate was also monitored by ICP-OES, illustrating that best separation is reached after 24h (Supplementary Table 11).

2.10 Extraction of lamp phosphor

This experiment was carried out in a well-ventilated fumehood.

A compact fluorescent light bulb (PHILIPS Genie 14 W energy saver 230-240 V) was crushed inside a plastic bag to separate the glass from the bulb socket. The extracted glass (21.150 g) was mortared into a fine powder. That powder was then suspended in a 1:2 mixture of trifluoromethanesulfonic acid (2 mL) in water at 0 °C, resulting in an off-white slurry. The suspension was heated at 110 °C for 2 h resulting in a color change to light pink. Water was added (10 mL) and the slurry was stirred for 30 min before being filtered and the filtrate taken

to dryness at 200 °C for 25 h (1.2275 g, 5.8 wt%) and was characterized by ICP-OES (Supplementary Table 12).

The process is visually illustrated in the Supplementary Movie 1.

2.11 Separation of Eu from lamp phosphor

In a 25 mL scintillation vial, $(NEt_4)_2WS_4$ (0.500 g, 0.87 mmol) was solubilized in 20 mL of MeCN resulting in a bright yellow solution. Lamp phosphor powder extracted according to the process described above (0.200 g) was added as a solid to this solution, resulting in an immediate color change to dark red. After one hour a golden-brown precipitate had formed. The reaction was stirred at room temperature for 24h, before the solution was centrifuged to separate the resulting golden-brown powder which was further washed with MeCN (2 x 4 mL) and dried under vacuum (24.6 mg, 12 wt%) and a red filtrate which was taken to dryness (659.4 mg, >3 wt% loss). Both phases were characterized by ICP-OES (Supplementary Table 13).

The process is visually illustrated in the Supplementary Movie 1.

2.12 Recovery of Europium oxide from complex 1

Complex 1 (100 mg, 0.096 mmol, 1 equiv.) was solubilized in 5 mL of degassed miliQ water resulting in a yellow solution. Ammonium oxalate (27 mg, 0.192 mmol, 2 equiv.) was added as a solid to the solution, leading immediately to the precipitation of a dark red-brown solid of europium oxalate. The reaction was stirred at room temperature for 1 h before the solution was centrifuged. The yellow supernatant was transferred to a Schlenk flask and taken to dryness (82 mg), and an aliquot was analyzed by UV-vis spectroscopy (Fig. S7), confirming the recovery of WS_4^{2-} in the aqueous phase. The red-brown solid of europium oxalate was washed with water (2 x 5 mL) and dried under vacuum (24.5 mg, 0.102 mmol), before being calcined in a quartz tube furnace at 600 °C for 2 h under a flow of dry air (150 mL/min). A white solid which exhibits red luminescence when excited with a UV lamp (395 nm) is obtained (15.3 mg, 0.043 mmol, 85 % yield). Elemental analysis found by ICP-OES (calc.) % for Eu_2O_3: Eu, 78.60 (86.36), corresponding to a 91% purity.

2.13 Recovery of Europium oxide from complex 1:1 (wt%) Eu:Y model mixture

The golden solid obtained from the 1:1 (wt%) Eu:Y separation from the model mixture (115 mg, 0.11 mmol, 1 equiv.) was solubilized in 5 mL of degassed miliQ water resulting in a yellow solution. Ammonium oxalate (40 mg, 0.28 mmol, 2.5 equiv.) was added as a solid to the solution, leading immediately to the precipitation of a dark red-brown solid. The reaction was stirred at room temperature for 1 h before the solution was centrifuged. The yellow supernatant was transferred to a Schlenk flask and taken to dryness. The red-brown solid was rinsed with water (2 x 10 mL) and dried under vacuum (25 mg, 0.102 mmol, 93 % yield assuming a composition as EuC_2O_4), before being calcined in a quartz tube furnace at 600 °C for 2 h under a flow of dry air (150 mL/min). This resulted in the formation of a white solid (10.3 mg, 0.03 mmol) Elemental analysis found (calc.) % for Eu_2O_3: Eu, 77.56 (86.36). Y 2.198 (0), corresponding to a 90% purity.

3. Cyclic Voltammetry

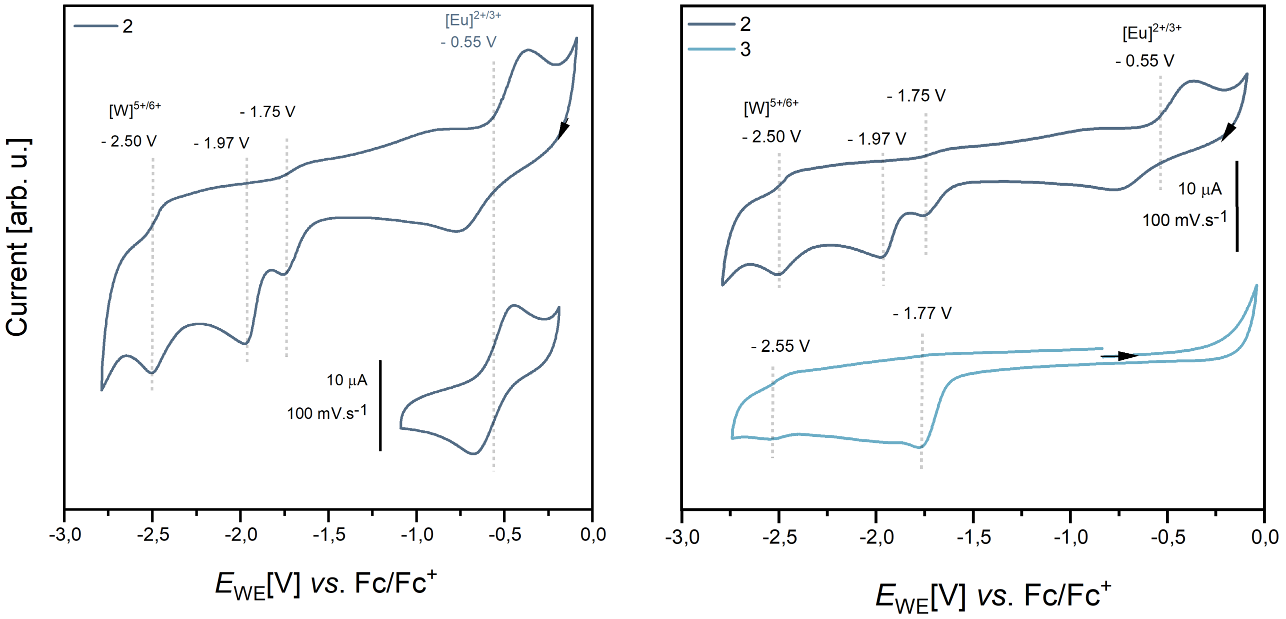

Supplementary Figure 1: CV spectra of complex 2 (dark blue) and 3 (light blue) (1 mM) in 0.1 M TBAPF6 in MeCN

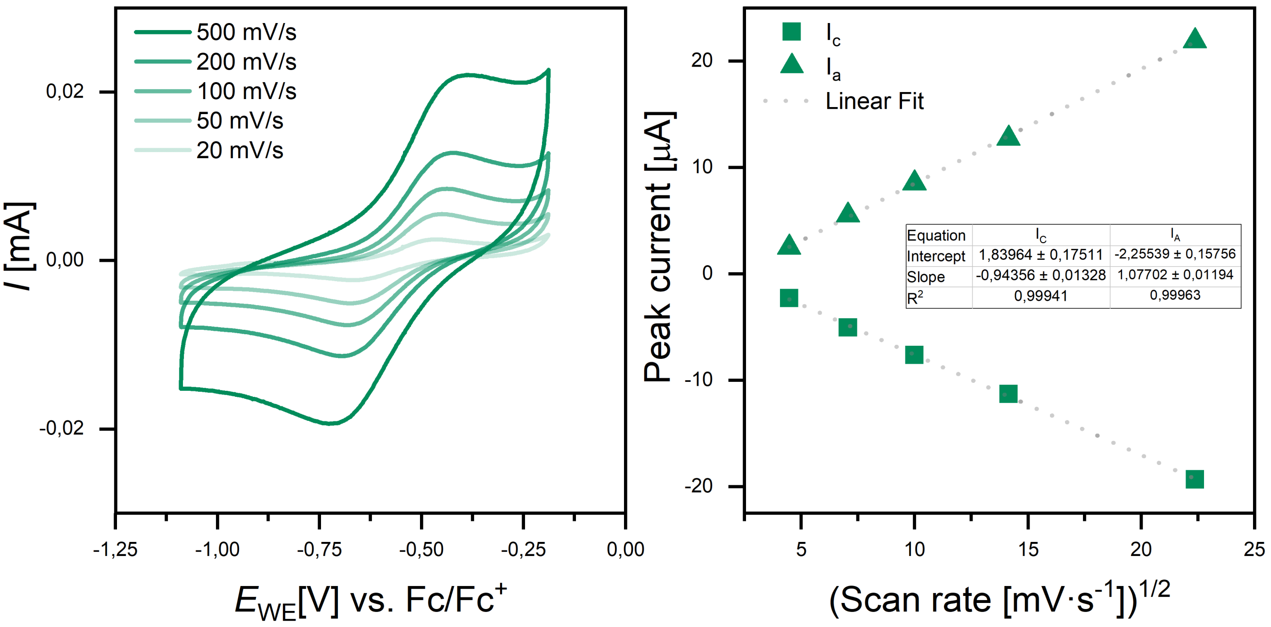

Supplementary Figure 2: CV spectra of Eu(III)/Eu(II) reduction wave in complex **2** (1 mM) in 0.1 M $TBAPF_6$ in MeCN at different scan rates (left); Peak current plotted against the square root of the scan rate for scan rate dependance CV measure for complex **2** (right), with cathodic peak current I_C (square) and anodic peak current I_A (triangle).

4. UV-Vis Spectroscopy

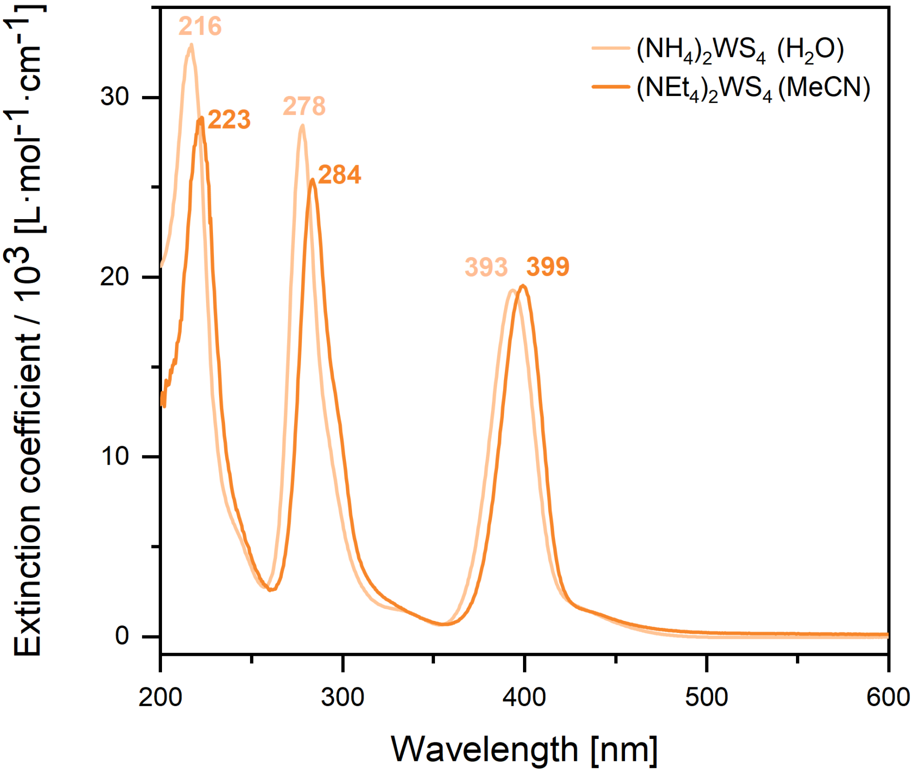

Supplementary Figure 3: UV-Vis electronic absorption spectrum of $(NH_4)_2WS_4$ in H_2O (light orange) and $(NEt_4)_2WS_4$ in MeCN (dark orange).

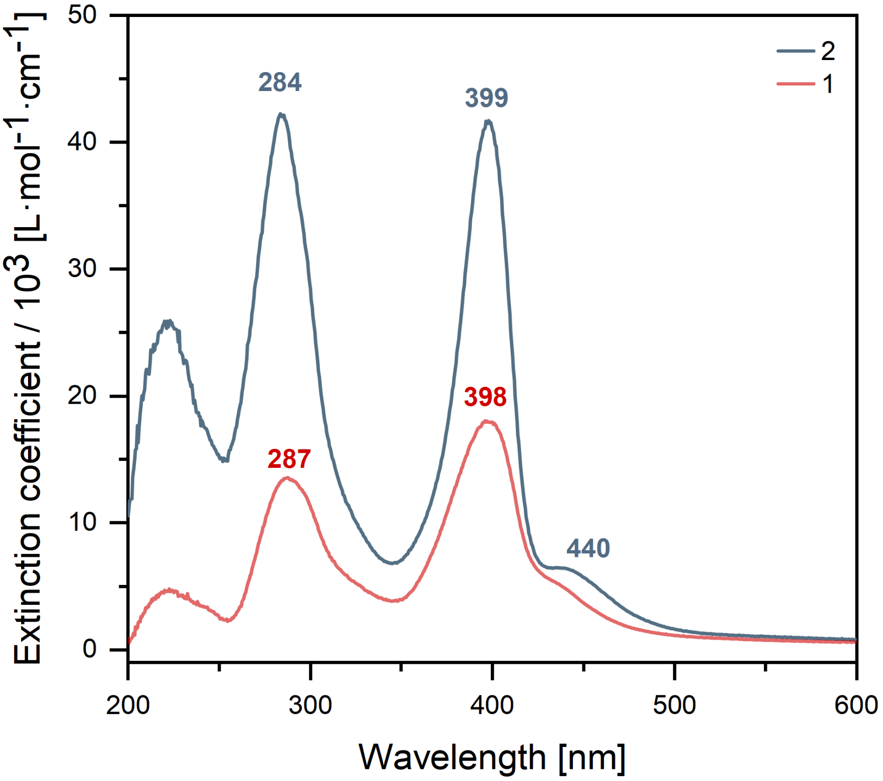

Supplementary Figure 4: UV-Vis electronic absorption spectrum of **1** (red) and **2** (blue) in MeCN.

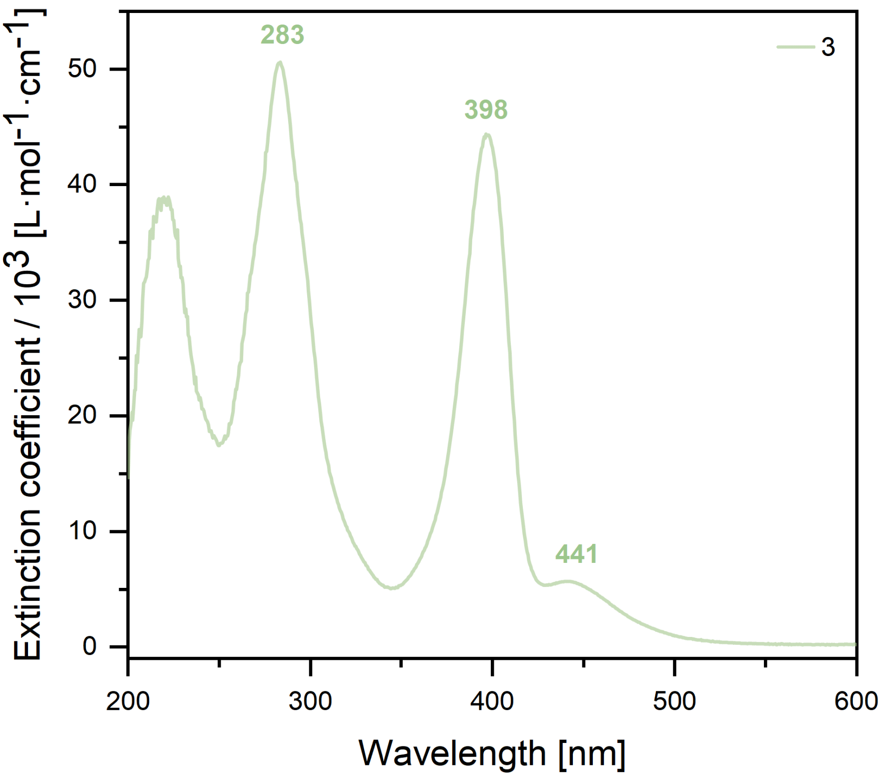

Supplementary Figure 5: UV-Vis electronic absorption spectrum of **3** (green) in MeCN.

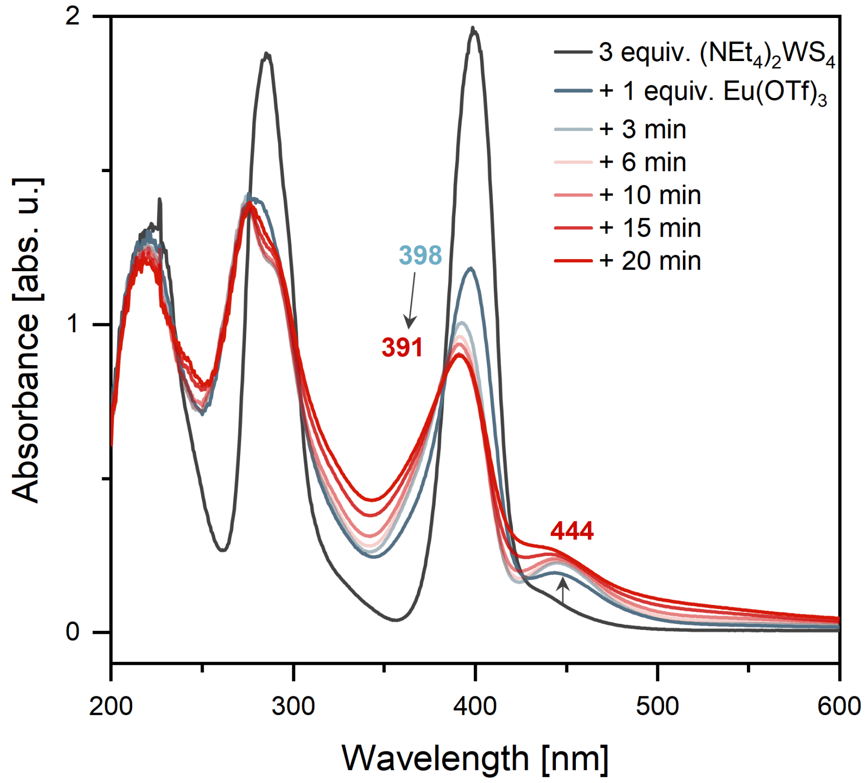

Supplementary Figure 6: UV-Vis electronic absorption spectrum of the formation of complex **1** (red) from $(NEt_4)_2WS_4$ (black), via the intermediate formation of 2 (blue).

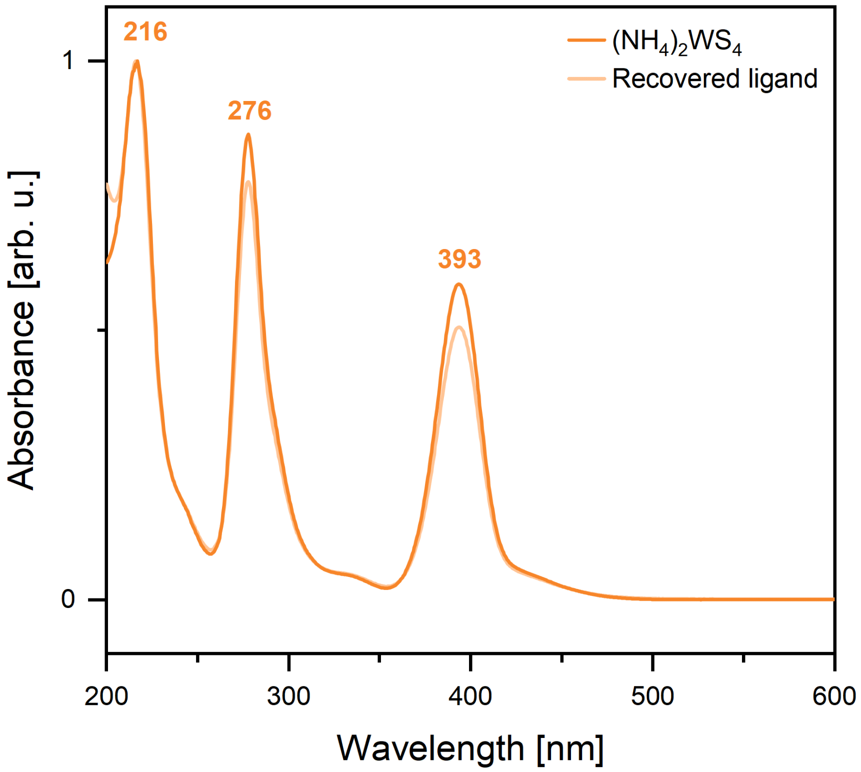

Supplementary Figure 7: UV-Vis electronic absorption spectrum of the $(NH_4)_2WS_4$ (prepared according to section 2.1 (dark orange) in H_2O and the aqueous supernatant obtained according to process 2.12 (light orange). Spectrum were normalized from the 216 nm band intensity.

5. Raman Spectroscopy

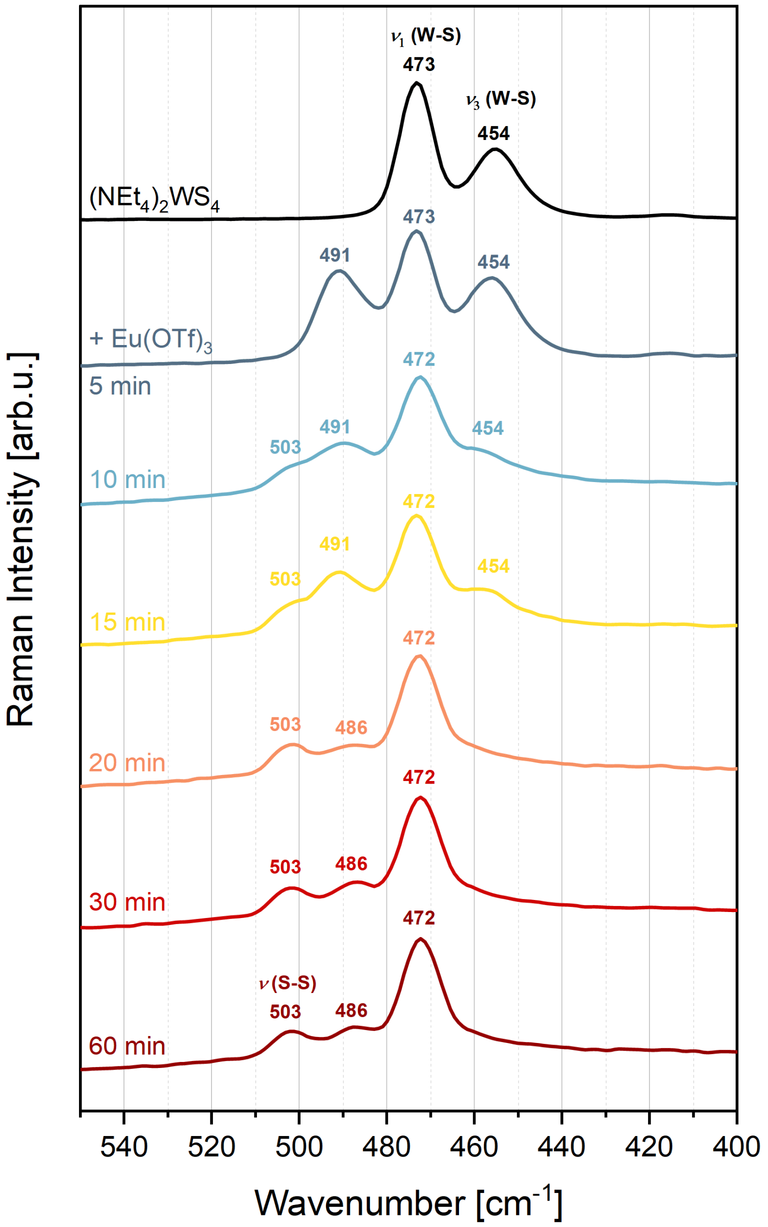

Supplementary Figure 8: Low-energy region of resonance Raman spectra recorded on solution samples at room temperature with 532 nm laser excitation.

6. Infrared Spectroscopy

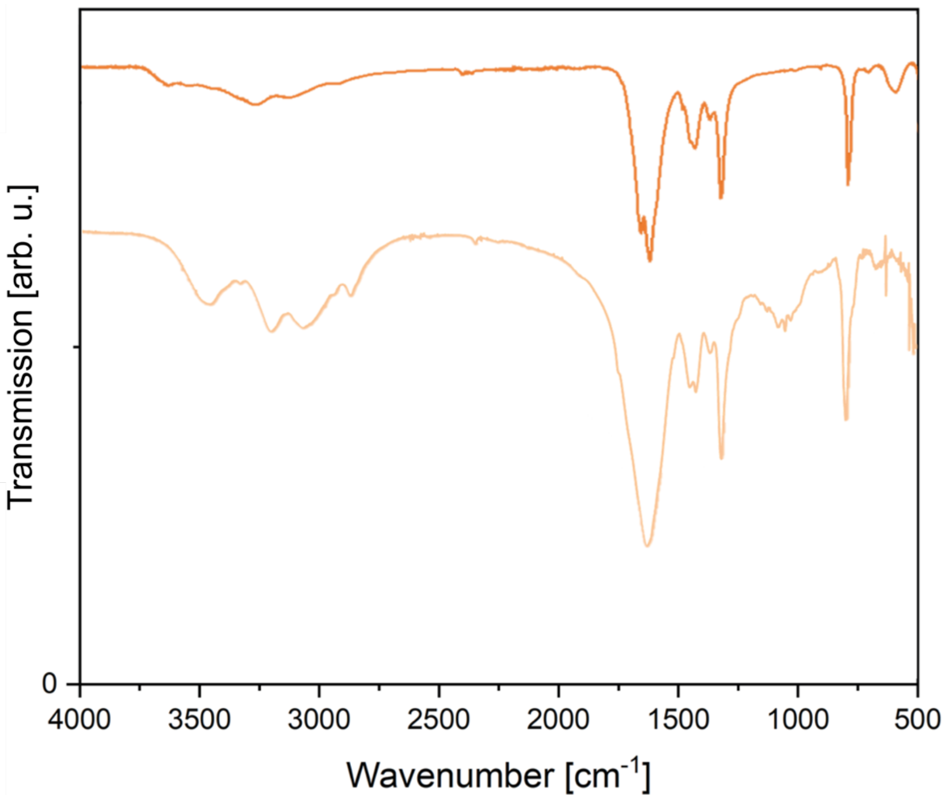

Supplementary Figure 9: FTIR spectra of europium oxalate: as prepared in this work according to section 2.12 (light orange) and from ref[15] (orange). Graph extracted from the Supplementary information section, Figure S59 therein).

7. X-Ray Photoelectron Spectroscopy

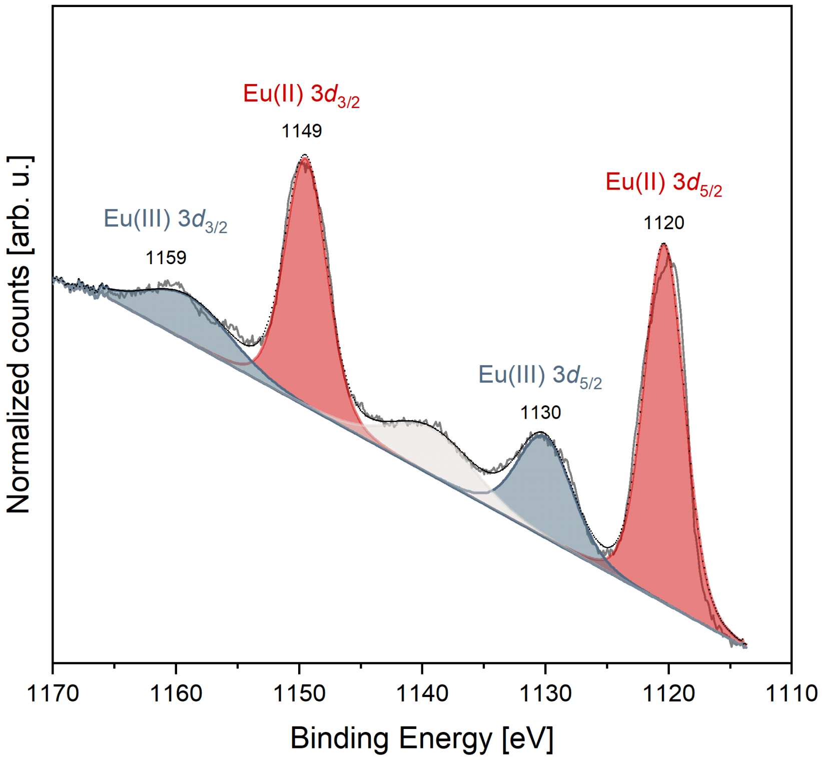

Supplementary Figure 10: XPS spectra in the Eu 3*d* region for complex **1.**

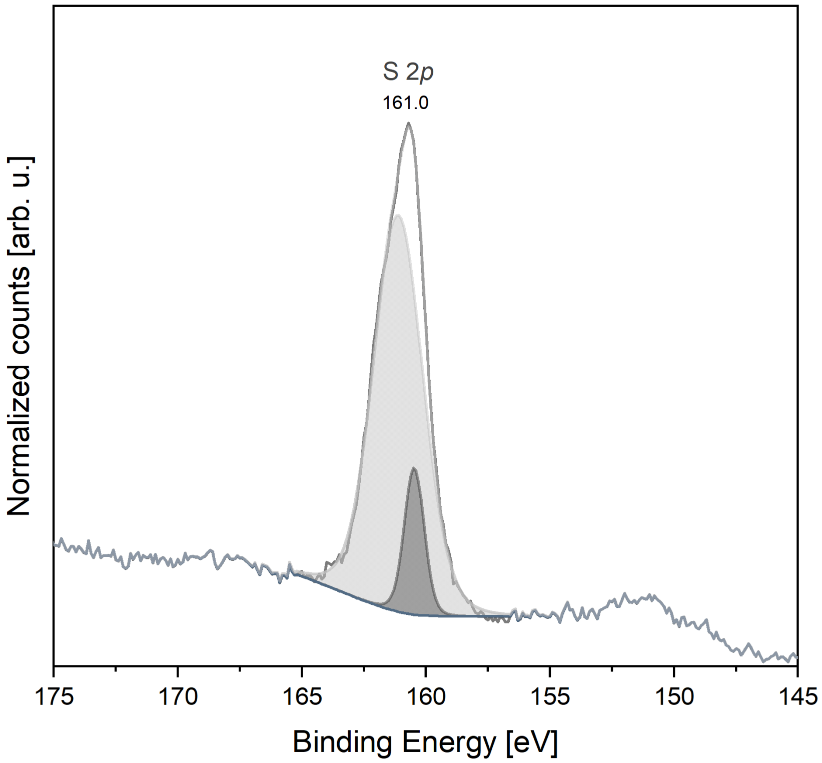

Supplementary Figure 11: XPS spectra in the S 2p region for complex 1.

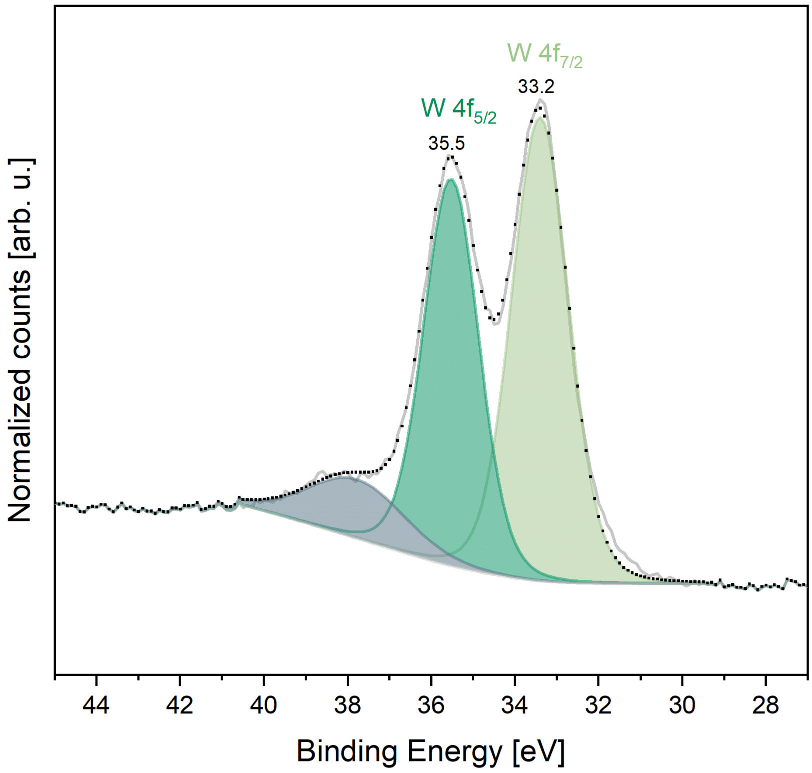

Supplementary Figure 12: XPS spectra in the W 4f region for complex 1.

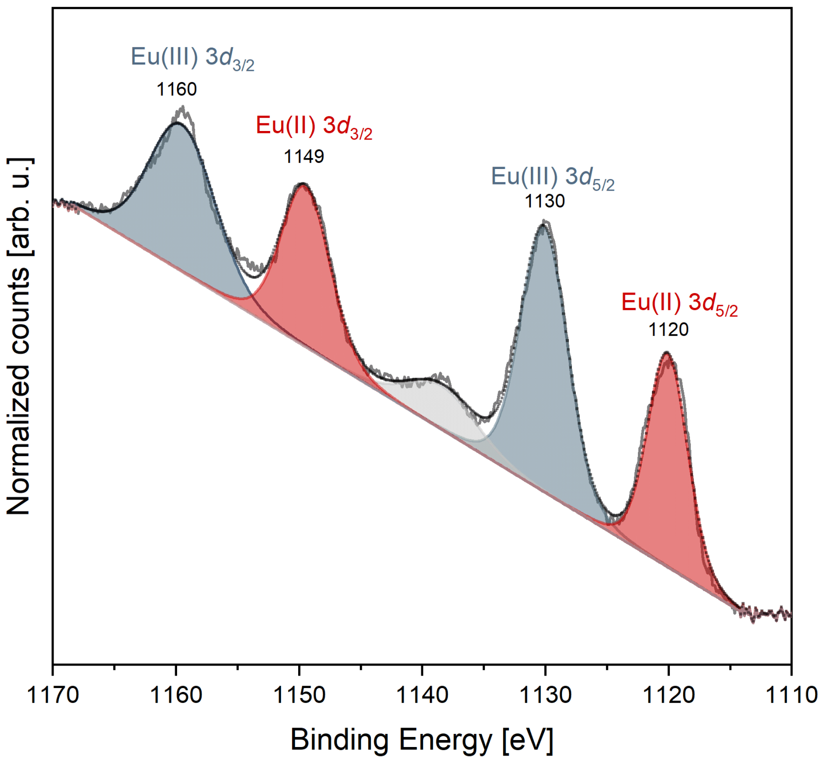

Supplementary Figure 13: XPS spectra in the Eu 3d region for complex 2.

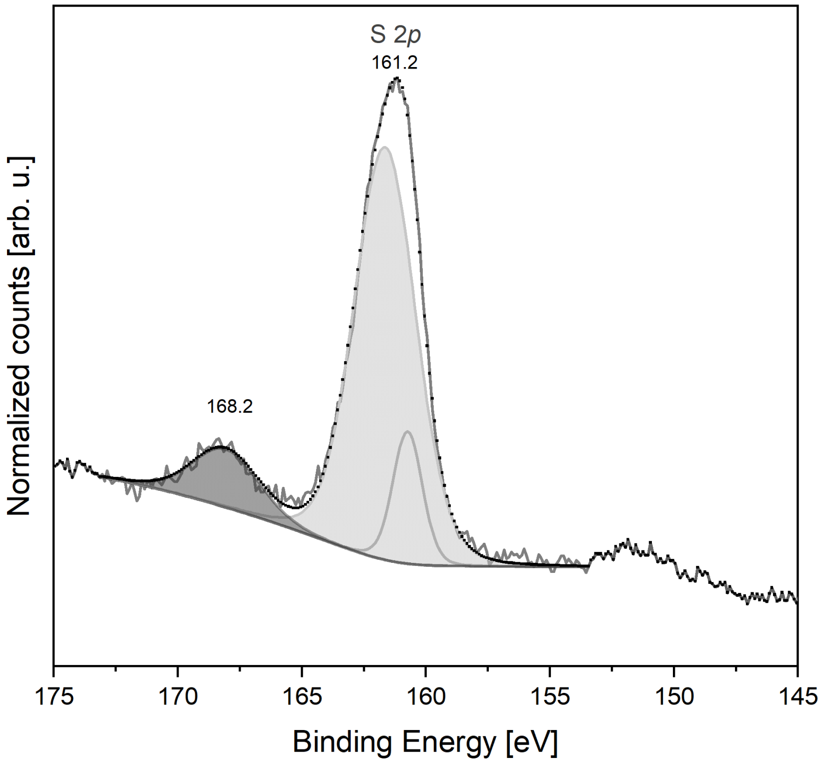

Supplementary Figure 14: XPS spectra in the S 2*p* region for complex **2.**

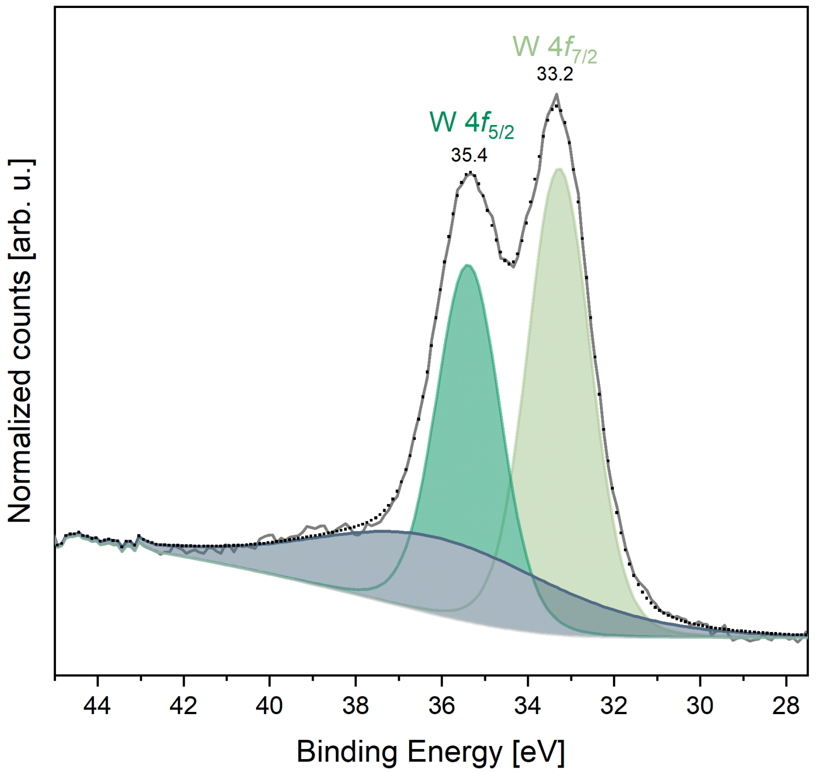

Supplementary Figure 15: XPS spectra in the W 4*f* region for complex **2.**

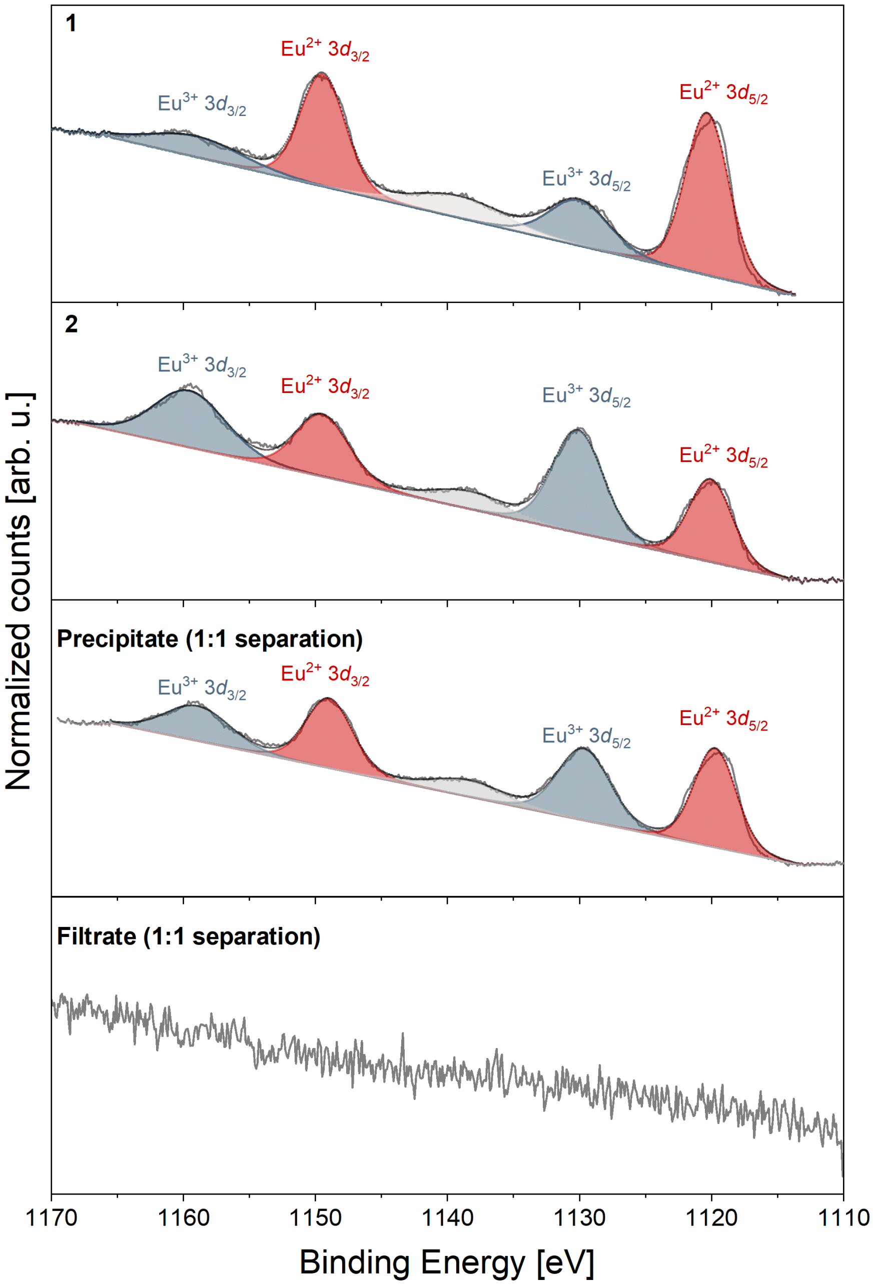

Supplementary Figure 16: XPS spectra in the Eu 3*d* region for complexes **1** and **2**, and for the precipitate and filtrate obtained from the 1:1 (wt%) mixture separation.

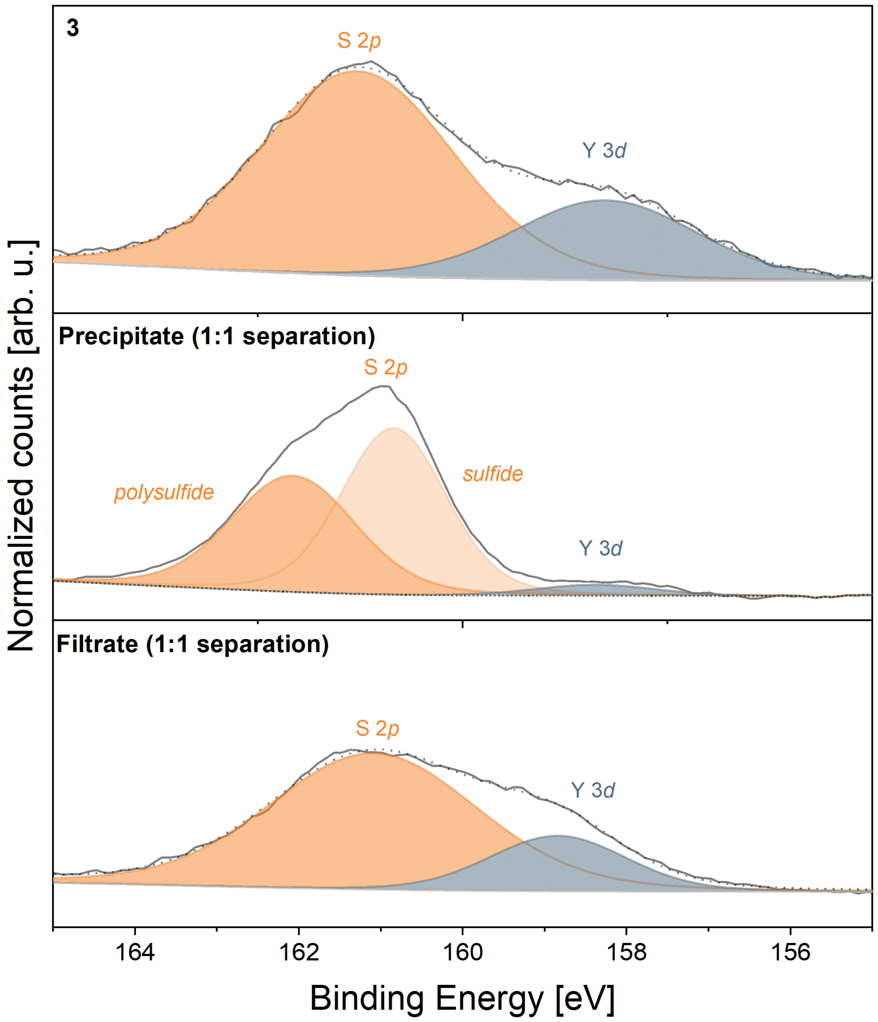

Supplementary Figure 17: XPS spectra in the Y 3*d* region for the filtrate of the synthesis of complex **1,** complex **3** and for the precipitate and filtrate obtained from the 1:1 (wt%) mixture separation.

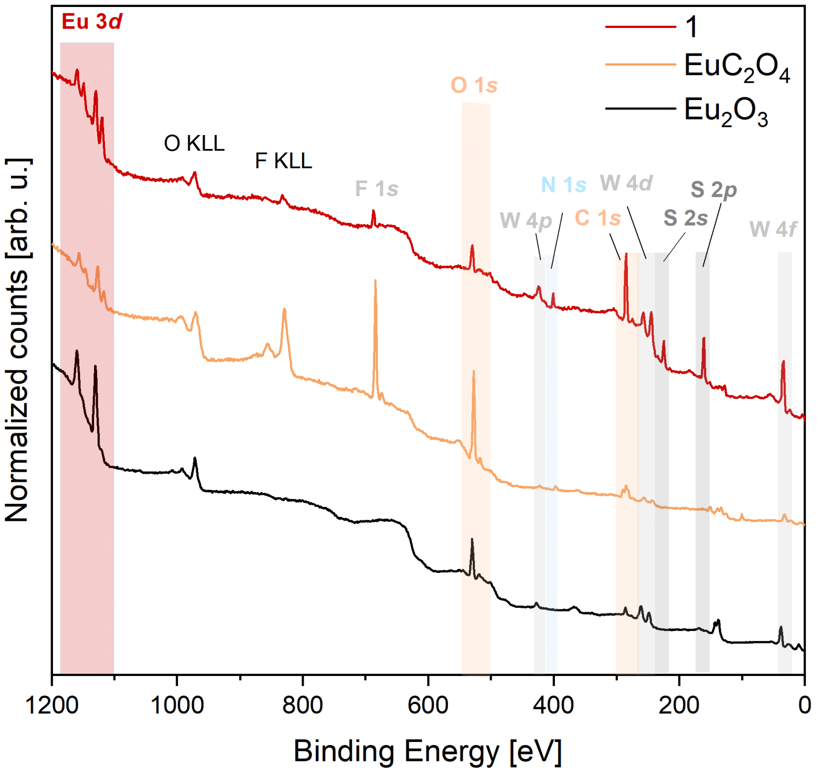

Supplementary Figure 18: XPS survey spectra of complex **1** (red), europium oxalate (orange) and europium oxide (black) prepared according to section 2.12. A strong F 1*s* peak can be observed in the spectra of the europium oxalate precipitate, and can be assigned to co-precipitated $[NEt_4]OTf$ by-product. Nevertheless, this signal vanishes after calcination of the oxide, highlighting that this by-product can be quantitatively thermally decomposed.

Supplementary Table 1: Selected binding energies (eV) of Eu 3*d* in complexes **1-2** and in europium oxalate and europium oxide as described in synthesis 2.12. For EuC_2O_4 and Eu_2O_3 the spectra were calibrated using the reported O 1*s* energy. Binding energy values for EuC_2O_4 and Eu_2O_3 are in good agreement with reported values, [16] and tungsten 4f binding energy values corroborate a +6 oxidation state.[17]

Structural parameters	Eu $3d_{3/2}$	Eu $3d_{5/2}$	S 2p	W $4f_{5/2}$	W $4f7_{/2}$	O 1s
**1**	1159.4<br>1149.6	1130.4<br>1120.4	161.0	35.5	33.3	529.5
**2**	1160.0<br>1149.7	1130.2<br>1120.0	161.2	35.4	33.2	530.3
**EuC_2O_4**	1161.2<br>1151.3	1132.0<br>11222.2	-	-	-	532.8<br>(532.8)
**Eu_2O_3**	1159.5	1130.2	-	-	-	530.0 (530)

8. X-Ray Absorption Spectroscopy

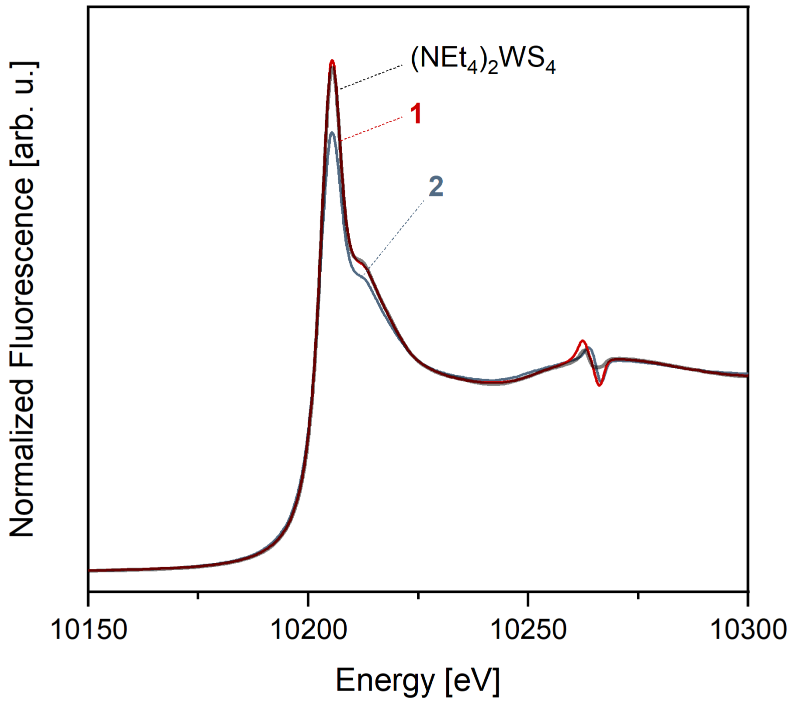

Supplementary Figure 19: Normalized XANES spectra measured in transmission mode at W L_3-edge for $(NEt_4)_2WS_4$ (black), complexes **1** (red) and **2** (blue). Identical whiteline energy can be determined for all three species.

9. Single Crystal X-Ray Diffraction

All structures reported herein have been deposited on the Cambridge Structural Database under CSD numbers 2312646, 2312677, 2312650 (See Supplementary Table 3).

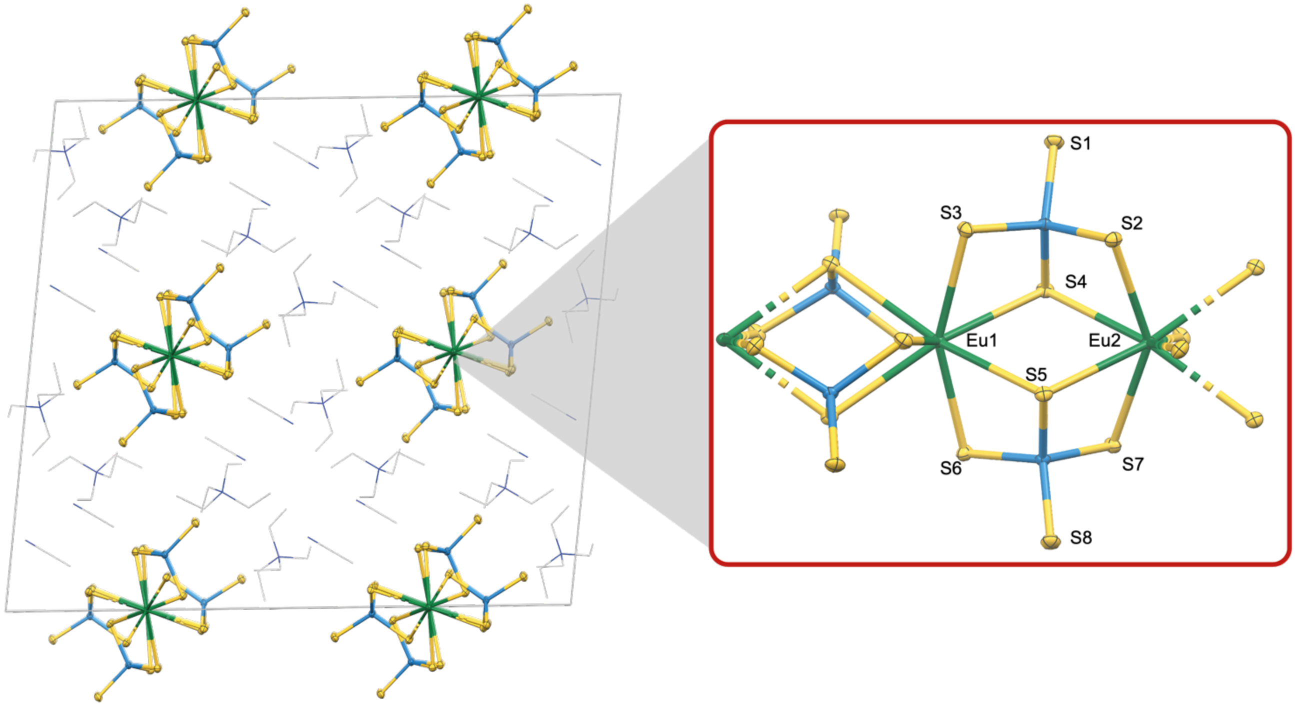

Supplementary Figure 20: Mercury diagram of the solid-state molecular structure of **1**. Hydrogen atoms have been omitted for clarity. ORTEP thermal ellipsoids are shown at the 50% probability level. Selected bond lengths and angles are summarized in Supplementary Table 2.

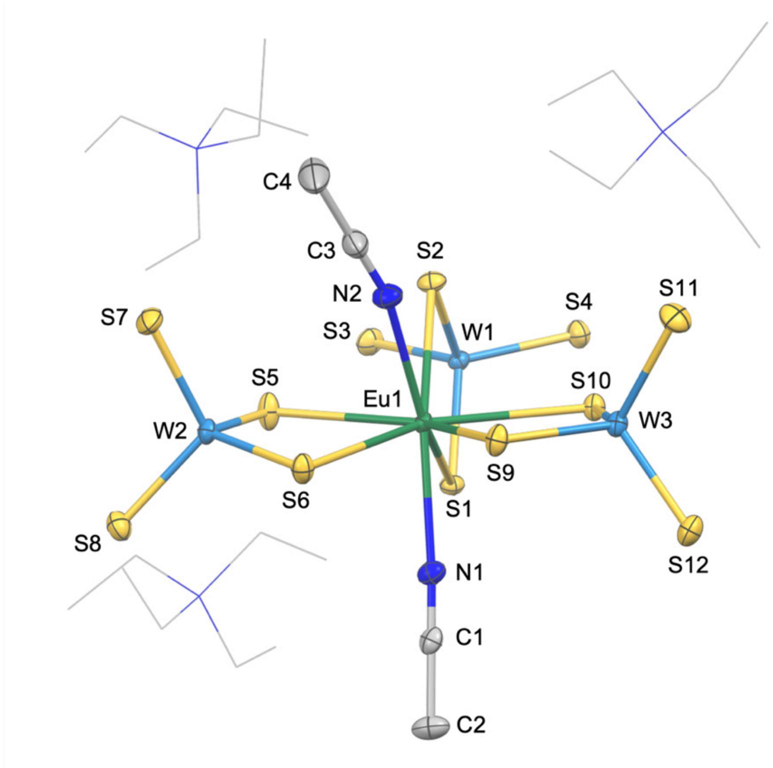

Supplementary Figure 21: Mercury diagram of the solid-state molecular structure of **2**. Hydrogen atoms and co-crystallized MeCN molecules have been omitted for clarity. ORTEP thermal ellipsoids are shown at the 50% probability level. Selected bond lengths and angles are summarized in Supplementary Table 2.

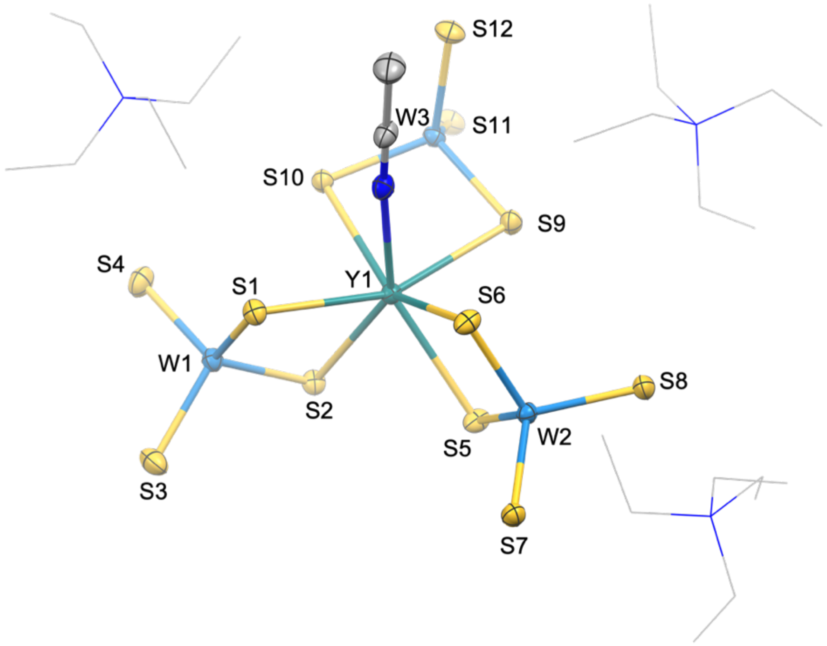

Supplementary Figure 22: Mercury diagram of the solid-state molecular structure of **3**. Hydrogen atoms and co-crystallized MeCN molecules have been omitted for clarity. ORTEP thermal ellipsoids are shown at the 50% probability level. Selected bond lengths and angles are summarized in Supplementary Table 2.

1 Alert level B: `PLAT971_ALERT_2_B Check Calcd Resid. Dens.   0.95Ang From W   2.53 eA-3`

This alert is unproblematic as residual electron density near heavy atoms is common.

Supplementary Table 2: Selected bond distances and angles for complexes **1-3**.

Structural parameters	**1**	Structural parameters	**2**	Structural parameters	**3**
Eu(1)-W(1)	3.7943(3)	Eu(1)-W(1)	3.6314(3)	Y(1)-W(1)	3.5183(4)
Eu(1)-W(2)	3.8175(3)	Eu(1)-W(2)	3.6620(3)	Y(1)-W(2)	3.5445(3)
Eu(1)-S(3)	2.9691(9)	Eu(1)-W(3)	3.6576(3)	Y(1)-W(3)	3.5256(4)
Eu(1)-S(4)	3.2063(10)	Eu(1)-S(1)	2.9216(11)	Y(1)-S(1)	2.7722(10)
Eu(1)-S(5)	3.1892(10)	Eu(1)-S(2)	2.9050(12)	Y(1)-S(2)	2.8465(10)
Eu(1)-S(6)	3.0310(10)	Eu(1)-S(5)	2.8970(12)	Y(1)-S(5)	2.8434(10)
Eu(2)-S(2)	3.0440(10)	Eu(1)-S(6)	2.9546(12)	Y(1)-S(6)	2.7753(10)
Eu(2)-S(4)	3.1704(10)	Eu(1)-S(9)	2.8958(12)	Y(1)-S(9)	2.7666(10)
Eu(2)-S(5)	3.1517(10)	Eu(1)-S(10)	2.8950(11)	Y(1)-S(10)	2.8452(10)
Eu(2)-S(7)	3.0305(10)	W(1)-S(1)	2.2062(11)	W(1)-S(1)	2.2329(9)
W(1)-S(1)	2.1716(9)	W(1)-S(2)	2.2063(10)	W(1)-S(2)	2.2156(9)
W(1)-S(2)	2.1894(10)	W(1)-S(3)	2.1751(11)	W(1)-S(3)	2.1595(10)
W(1)-S(3)	2.2019(10)	W(1)-S(4)	2.1761(12)	W(1)-S(4)	2.1650(11)
W(1)-S(4)	2.2073(10)	W(2)-S(5)	2.2045(12)	W(2)-S(5)	2.2135(10)
W(2)-S(5)	2.2097(10)	W(2)-S(6)	2.2101(11)	W(2)-S(6)	2.2128(10)
W(2)-S(6)	2.1903(9)	W(2)-S(7)	2.1807(12)	W(2)-S(7)	2.1717(10)
W(2)-S(7)	2.1950(9)	W(2)-S(8)	2.1715(12)	W(2)-S(8)	2.1674(10)
W(2)-S(8)	2.1747(10)	W(3)-S(9)	2.2103(11)	W(3)-S(9)	2.2304(10)
-	-	W(3)-S(10)	2.2039(11)	W(3)-S(10)	2.2087(8)
-	-	W(3)-S(11)	2.1780(13)	W(3)-S(11)	2.1688(10)
-	-	W(3)-S(12)	2.1782(12)	W(3)-S(12)	2.1707(10)

Supplementary Table 3: Refinement parameters for complexes **1-3**.

	**1**	**2**	**3**
Formula	$[NEt_4]_2[Eu^{II}(WS_4)_2]\cdot 2(MeCN)$	$lEt_4]_3[Eu^{III}(MeCN)_2(WS_4)_3]\cdot MeCN$	$[NEt_4]_3[Y^{III}(MeCN)_2(WS_4)_3]$
Empirical formula	$C_{20}H_{46}EuN_4S_8W_2$	$C_{30}H_{69}EuN_6S_{12}W_3$	$C_{28}H_{66}N_5S_{12}W_3Y$
Molecular weight [g/mol]	1118.75	1602.14	1498.03
Temperature [k]	100	100	100
Crystal system	*monoclinic*	*monoclinic*	*monoclinic*
Space group	*C*2/*c*	*P*2_1/*c*	*P*2_1/*c*
a [å]	25.1261(4)	15.25930(10)	19.28810(10)
b [å]	10.2348(1)	11.39130(10)	13.92450(10)
c [å]	27.6163(4)	35.14130(10)	20.0351(2)
α [°]	90	90	90
β [°]	96.270(1)	118.3240(10)	109.8900(10)
γ [°]	90	90	90
V [$Å^3$]	7059.34(17)	5378.16(9)	5059.98(7)
Z	8	4	4
ρ_{calc} [$g\cdot cm^{-3}$]	2.105	1.979	1.966
μ [mm^{-1}]	28.884	24.344	18.598
F(000)	4264.0	3072.0	2888.0
Crystal size [mm^3]	0.03 × 0.03 × 0.14	0.11× 0.052 × 0.026	0.359 × 0.096 ×0.048
Radiation	CuKα	CuKα	CuKα
2θ range [°]	6.44 to 160.614	5.712 to 156.59.8	4.872 to 160.442
Reflections collected	28907	52737	43366
Independent reflexions	7477	11342	10807
Final r indexes [i>=2σ (i)]	R1 = 0.0264, wR2 = 0.0705	R1 = 0.0242, wR2 = 0.0560	R1 = 0.0310, wR2 = 0.0871
Final r indexes [all data]	R1 = 0.0304, wR2 = 0.0727	R1 = 0.0253, wR2 = 0.0566	R1 = 0.0328, wR2 = 0.0884
Goof f^2	1.074	1.048	1.100
Largest diff. Peak/hole [$e\cdot Å^{-3}$]	0.97/–0.89	1.19/–1.15	2.47/–1.32
CSD number	2312650	2312646	2312677

10. Pair Distribution Function Analysis

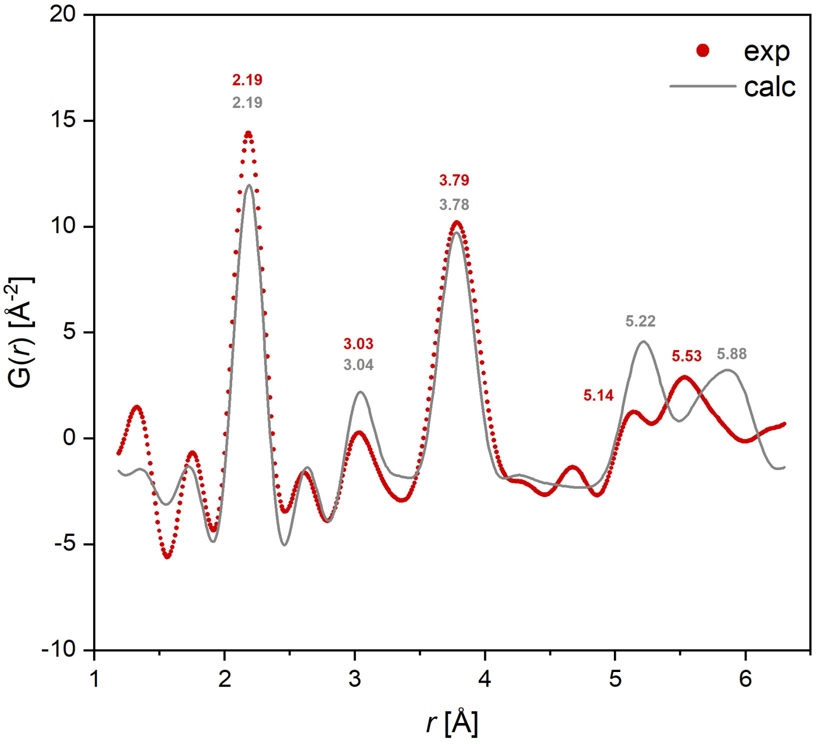

Supplementary Figure 23: The calculated X-ray pair distribution function G(r) of **1** in the distance interval 1.2-6.3 Å based on single crystal XRD data (grey line) and the experimental PDF of the golden precipitate (red dots). The peaks at 2.19 Å, 3.03 Å and 3.79 Å, correspond to the next neighbor W-S, Eu-S and W-Eu distances (2.19 Å, 3.04 Å and 3.78 Å, respectively) found in the PDF of compound **1**.

11. Bond Valence Sum Analysis

The oxidation state for each europium atom was calculated using the formula:

$$V_i = \sum s_{ij}$$

Considering the bond valence for the atom i as defined by Brown[18]:

$$s_{ij} = exp[\frac{R0 - r_{ij}}{B}]$$

Where r_{ij} is the experimental bond length while R0 and B are empirically determined constants for a given i-j bond, for all Eu-X bonds in complex **1**.

While the published parameters for Eu-S bonds[19] (R0=2.584 and B=0.37) for Eu-S bonds give a consistent oxidation state assignment for Eu as Eu(II), the published parameters[20] for W-S bonds (R0=2.39 and B=0.37) did not enable to model appropriately even the starting $[NEt_4]WS_4$ complex, as resulting in a bond valence sum of 6.8. We redetermined the R0 parameter to obtain a valence of 6.00 for the reference species $[NEt_4]WS_4$, and calculated a value of R0 = 2.342

Supplementary Table 4. Bond valence sum for Eu in **1**.

Atom	Eu(1)	Eu(2)
V	2.06	2.01

Supplementary Table 5. Bond valence sum for W in **1**.

Atom	W(1)	W(2)
V	6.04	6.04

Supplementary Table 6. Bond valence sum for W in **2**.

Atom	W(1)	W(2)	W(3)
V	6.02	6.00	5.99

Supplementary Table 7. Bond valence sum for W in **3**.

Atom	W(1)	W(2)	W(3)
V	6.00	6.02	5.97

12. Eu/Y Separation

12.1 Determination of Enrichment, Separation Factors and Efficiency

The separation factor $S_{Eu/Y}$ was determined by the following equation:

$$S_{Eu/Y} = D_{solid} \times D_{filtrate} = \frac{\eta_{Eu}}{\eta_Y} \times \frac{\eta_Y}{\eta_{Eu}}$$

Where the weight ratios (wt%) were determined by ICP-OES spectroscopy.

The recovery efficiency was determined as the percentage of Europium extracted from the original mixture using the following equation:

$$R_{Eu} = 100 - \left[\frac{C_{Eu-liquid}}{C_{Eu-imput}} \times 100\right]$$

Where the concentrations (ppm) were determined by ICP-OES spectroscopy.

12.2 Separation of Eu from 1:1 Eu/Y model mixture

Supplementary Table 8. ICP-OES quantification of the Eu/Y quantification in the precipitate (Eu phase) and filtrate (Y phase) from the 1:1 wt% $Eu(OTf)_3$ and $Y(OTf)_3$ mixture and duplicates.

Entry	WS_4 extractant	Solid phase (wt%)	Liquid phase (wt%)	Separation factor $S_{Eu/Y}$	Recovery Efficiency R_{Eu}
1	1:1 $Eu(OTf)_3$:$Y(OTf)_3$	11.336 ± 0.115 (Eu) 0.241 ± 0.003 (Y)	0.052 ± 0.001 (Eu) 2.553 ± 0.022 (Y)	2308.4 ± 27.5	94.8
2	1:1 $Eu(OTf)_3$:$Y(OTf)_3$	13.710 ± 0.137 (Eu) 0.279 ± 0.003 (Y)	0.025 ± 0.001 (Eu) 2.462 ± 0.019 (Y)	4857.9 ± 59.6	97.5

Supplementary Table 9. Separation factor from the 1:1 (wt%) Eu:Y model mixtures with different extractants.

Reference	Method	Extractant	Extraction steps	Eu recovery efficiency (%)	Separation factor $S_{Eu/Y}$
Binnemans et al. (2020)[21]	liquid-liquid (organic only)	Cyanex 923	1		46
Schelter et al. (2016)[22]	Solid-liquid extraction	TriNOx	1		39.2
Schelter et al. (2017)[23]	Electro-kinetic separation (redox + solid-liquid extraction)	TriNOx	1		74.8
**This work**	**IIET**	**$(NEt_4)_2WS4$**	**1**	**96.2***	**3583***

*average value from duplicate experiments – see table S8

12.3 Separation of Eu from 1:14 Eu/Y model mixture

Supplementary Table 10. ICP-OES quantification of the Eu/Y quantification in the precipitate (Eu phase) and filtrate (Y phase) from the 1:14 wt% $Eu(OTf)_3$ and $Y(OTf)_3$ mixture and duplicates.

Entry	WS4 extractant	Solid phase (wt%)	Liquid phase (wt%)	**Separation factor $S_{Eu/Y}$**	**Recovery Efficiency R_{Eu}**
1	1:14 $Eu(OTf)_3$:$Y(OTf)_3$	11.338 ± 0.115 (Eu)<br>0.814 ± 0.08 (Y)	0.018 ± 0.000 (Eu)<br>3.718 ± 0.024 (Y)	2926.4 ± 18.4	98.2
2	1:14 $Eu(OTf)_3$:$Y(OTf)_3$	9.973 ± 0.217 (Eu)<br>1.286 ± 0.028 (Y)	0.011 ± 0.000 (Eu)<br>3.999 ± 0.029 (Y)	2848.7 ± 30.2	98.9

Supplementary Table 11. ICP-OES quantification of the precipitate (Eu phase) over time from the 1:14 wt% $Eu(OTf)_3$ and $Y(OTf)_3$ mixture and duplicates.

	2 h	18 h	24 h
Composition (wt%)	7.632 ± 0.601 (Eu)<br>0.804 ± 0.022 (Y)	9.321 ± 0.444 (Eu)<br>0.504 ± 0.024 (Y)	12.909 ± 0.162(Eu)<br>0.489 ± 0.006 (Y)

12.4 Extraction of lamp phosphor

Supplementary Table 12. ICP-OES quantification of the Eu and Y in the extracted lamp phosphor (PHILIPS Genie compact fluorescent light bulb (14 W energy saver 230-240 V)).

Lamp extraction	Eu (wt%)	Eu (ppm)	Y (wt%)	Y (ppm)	Y:Eu ratio
1	0.93	9268	12.6	125 597	13.6

12.5 Separation of Eu from lamp phosphor

Supplementary Table 13. ICP-OES quantification of the Eu/Y quantification in the precipitate (Eu phase) and filtrate (Y phase) from lamp extracts obtained according to the process described in section 2.11.

Entry	Solid phase (wt%)	D_{solid}	Liquid phase (wt%)	D_{liquid}	Eu (ppm)	**Separation factor $S_{Eu/Y}$**	**Recovery Efficiency R_{Eu}**
1	6.809 ± 0.103 (Eu)<br>2.540 ± 0.038 (Y)	2.68	0.012 ± 0.000 (Eu)<br>3.548 ± 0.028 (Y)	304.03	117	814.87 ± 13.5	98.7
2	7.468 ± 0.075 (Eu)<br>2.743 ± 0.028 (Y)	2.72	0.008 ± 0.000 (Eu)<br>3.803 ± 0.028 (Y)	466.54	82	1270.2 ± 7.4	99.1

B. Supplementary References

1 McDonald, J. W., Friesen, G. D., Rosenhein, L. D. & Newton, W. E. Syntheses and characterization of ammonium and tetraalkylammonium thiomolybdates and thiotungstates. *Inorganica Chim. Acta* **72**, 205-210 (1983). https://doi.org/https://doi.org/10.1016/S0020-1693(00)81720-X

2 Fairley, N. *et al.* Systematic and collaborative approach to problem solving using X-ray photoelectron spectroscopy. *Appl. Surf. Sci.* **5**, 100112 (2021). https://doi.org/https://doi.org/10.1016/j.apsadv.2021.100112

3 Muller, O., Nachtegaal, M., Just, J., Lutzenkirchen-Hecht, D. & Frahm, R. Quick-EXAFS setup at the SuperXAS beamline for in situ X-ray absorption spectroscopy with 10 ms time resolution. *J. Synchrot. Radiat.* **23**, 260-266 (2016). https://doi.org/https://doi.org/10.1107/S1600577515018007

4 Clark, A. H., Imbao, J., Frahm, R. & Nachtegaal, M. ProQEXAFS: a highly optimized parallelized rapid processing software for QEXAFS data. *J. Synchrot. Radiat.* **27** (2020). https://doi.org/https://doi.org/10.1107/S1600577519017053

5 Newville, M. IFEFFIT : interactive XAFS analysis and FEFF fitting. *J. Synchrot. Radiat.* **8**, 322-324 (2001). https://doi.org/10.1107/S0909049500016964

6 Ravel, B. & Newville, M. ATHENA, ARTEMIS, HEPHAESTUS: data analysis for X-ray absorption spectroscopy using IFEFFIT. *J. Synchrot. Radiat.* **12**, 537-541 (2005). https://doi.org/https://doi.org/10.1107/S0909049505012719

7 Sheldrick, G. M. A short history of SHELX. *Acta Crystallogr A* **64**, 112-122 (2008). https://doi.org/10.1107/s0108767307043930

8 Sheldrick, G. M. Crystal structure refinement with SHELXL. *Acta Crystallogr C Struct Chem* **71**, 3-8 (2015). https://doi.org/10.1107/s2053229614024218

9 Sheldrick, G. M. SHELXT - integrated space-group and crystal-structure determination. *Acta Crystallogr. A* **71**, 3-8 (2015). https://doi.org/https://doi.org/10.1107/S2053273314026370

10 Dolomanov, O. V., Bourhis, L. J., Gildea, R. J., Howard, J. A. K. & Puschmann, H. OLEX2: a complete structure solution, refinement and analysis program. *Journal of Applied Crystallography* **42**, 339-341 (2009).

11 Yang, X., Juhas, P., Farrow, C. L. & Billinge, S. J. L. xPDFsuite: an end-to-end software solution for high throughput pair distribution function transformation, visualization and analysis. *arXiv* (2014). https://doi.org/https://doi.org/10.48550/arXiv.1402.3163

12 Farrow, C. L. *et al.* PDFfit2 and PDFgui: computer programs for studying nanostructure in crystals. *J. Condens. Matter Phys.* **19**, 335219 (2007). https://doi.org/https://doi.org/10.1088/0953-8984/19/33/335219

13 Kobayashi, S. & Hachiya, I. Lanthanide Triflates as Water-Tolerant Lewis Acids. Activation of Commercial Formaldehyde Solution and Use in the Aldol Reaction of Silyl Enol Ethers with Aldehydes in Aqueous Media. *J. Org. Chem.* **59**, 3590-3596 (1994). https://doi.org/10.1021/jo00092a017

14 Carré, D. *et al.* Ternary sulfides formed by plurivalent europium with other metals. *J. less-common met.* **110**, 349-370 (1985). https://doi.org/https://doi.org/10.1016/0022-5088(85)90343-1

15 Huskić, I., Arhangelskis, M. & Friščić, T. Solvent-free ageing reactions of rare earth element oxides: from geomimetic synthesis of new metal–organic materials towards a simple, environmentally friendly separation of scandium. *Green Chem.* **22**, 4364-4375 (2020). https://doi.org/https://doi.org/10.1039/D0GC00454E

16 Uwamino, Y., Ishizuka, T. & Yamatera, H. X-ray photoelectron spectroscopy of rare-earth compounds. *J. Electron Spectrosc. Relat. Phenom.* **34**, 67-78 (1984). https://doi.org/https://doi.org/10.1016/0368-2048(84)80060-2

17 Sakane, G., Shibahare, T., Hou, H. W., Xin, X. Q. & Shi, S. The hexagonal prism shaped cluster [W2Ag4S8(AsPh3)4]: synthesis, crystal structure, and nonlinear optical properties. *Inorg. Chem.* **34**, 4785-4789 (1995). https://doi.org/https://doi.org/10.1021/ic00123a011

18 Brown, I. D. *The chemical bond in inorganic chemistry: the bond valence model*. Vol. 27 (Oxford university press, 2016).

19 Brown, I. D. <https://www.iucr.org/resources/data/datasets/bond-valence-parameters> (

20 Poisot, M., Näther, C. & Bensch, W. Synthesis, Spectroscopic and X-Ray Structure Characterisation of Bis(tetramethylammonium), Bis(tetraethylammonium) and Bis(tetrapropylammonium) Tetrathiotungstates. **61**, 1061-1066 (2006). https://doi.org/doi:10.1515/znb-2006-0903

21 Batchu, N. K., Dewulf, B., Riaño, S. & Binnemans, K. Development of a solvometallurgical process for the separation of yttrium and europium by Cyanex 923 from ethylene glycol solutions. *Sep. Purif. Technol.* **235**, 116193 (2020). https://doi.org/https://doi.org/10.1016/j.seppur.2019.116193

22 Bogart, J. A. *et al.* Accomplishing simple, solubility-based separations of rare earth elements with complexes bearing size-sensitive molecular apertures. *Proc. Natl. Acad. Sci. U.S.A.* **113**, 14887-14892 (2016). https://doi.org/10.1073/pnas.1612628113

23 Fang, H. *et al.* Electro-kinetic Separation of Rare Earth Elements Using a Redox-Active Ligand. *Angew. Chem. Int. Ed.* **56**, 13450-13454 (2017). https://doi.org/https://doi.org/10.1002/anie.201706894
