# Supplementary material for: Recovery of europium from E-waste using redox active tetrathiotungstate ligands
Source: Nat Commun. 2024 Jun 3;15:4577. doi: 10.1038/s41467-024-48733-z (PMC11148158; doi:10.1038/s41467-024-48733-z)
Supplement: Supplementary file 3 — Description of Additional Supplementary Files [file 41467_2024_48733_MOESM3_ESM.pdf]

Description of Additional Supplementary Files

File Name: Supplementary Movie 1
Description: Recovery of Europium from a spent fluorescent lamp.
